# Promicromonospora noduliphila sp. nov., a nodulation-enhancing actinobacterium isolated from the root nodules of grey-hair acacia planted in the Khurais desert, Saudi Arabia

**DOI:** 10.1099/ijsem.0.007173

**Published:** 2026-06-09

**Authors:** Khulud Alghannam, Grégoire Michoud, Alan Barozzi, Sarah Al Romaih, Rawan Alhazmi, Bob Vernooij, Kennedy Odokonyero, Adair Gallo, Himanshu Mishra, Daniele Daffonchio, Ramona Marasco

**Affiliations:** 1 Biological and Environmental Sciences and Engineering Division (BESE), King Abdullah University of Science and Technology (KAUST), Thuwal, Saudi Arabia; 2 River Ecosystems Laboratory, Alpine and Polar Environmental Research Centre, ENAC, Ecole Polytechnique Fédérale de Lausanne, Sion, Switzerland; 3 Environmental Science and Engineering (EnSE) Program, King Abdullah University of Science and Technology (KAUST), 23955-6900, Thuwal, Saudi Arabia; 4 Department of Agriculture, Forestry and Food Sciences (DISAFA), University of Turin, Grugliasco, Turin, Italy

**Keywords:** *Acacia gerrardii*, *Actinomycetota*, desert, *Promicromonosporaceae*, root nodules, symbiosis

## Abstract

A set of actinobacterial strains was isolated from the root nodules of *Acacia gerrardii* grown as part of an afforestation trial in the Khurais desert, Saudi Arabia. Among them, five clonal isolates could not be unambiguously assigned to any recognized type species, and two of them, designated AC027S^T^ and AC027N, were selected as representatives for taxonomic characterization. Cells were Gram-stain-positive, aerobic, non-motile, non-spore-forming, with short rods to coccoid-like morphology. Growth occurred at 20–42 °C, pH 5.0–10.0 and in the presence of up to 3.0% (w/v) NaCl. The major whole-cell sugar was glucose, and the muramic acid residues of the peptidoglycan carried peptide subunits composed of alanine, glutamic acid and lysine. The predominant menaquinone was MK-9(H4). Major fatty acids were iso-C_15:0_ and anteiso-C_15:0_, and the polar lipid profile included diphosphatidylglycerol, phosphatidylglycerols and several unidentified glycophospholipids, glycolipids, phospholipids and lipids. Phylogenetic analysis based on 16S rRNA gene sequences placed AC027S^T^ and AC027N within the genus *Promicromonospora*. Whole-genome sequence-based phylogenomic reconstruction and relatedness indices confirmed that these G+C-rich strains (72%) are distinct from all described *Promicromonospora* species, with *Promicromonospora soli* NEAU-GS50^T^ as the closest described relative. Consistent with their nodule-associated niche, the strains were able to degrade and utilize plant-derived substrates, tolerate microaerophilic conditions and possessed genes for mobilizing essential metals, such as iron and molybdenum, suggesting a supportive role in the nodule microbial community and in sustaining rhizobial nitrogen fixation, as reflected in an enhanced nodulation by native rhizobia when inoculated in desert soil. Based on phenotypic, chemotaxonomic and genomic evidence, strains AC027S^T^ and AC027N represent a novel species of the genus *Promicromonospora*, for which the name *Promicromonospora noduliphila* sp. nov. is proposed. The type strain is AC027S^T^ (=KCTC 59476^ᵀ^, =JCM 37759ᵀ).

## Data Summary

The 16S rRNA gene sequences of *Promicromonospora noduliphila* strains AC027S^T^ and AC027N have been deposited in the National Center for Biotechnology Information (NCBI) under the accession numbers PV257722 and PV257723, respectively. The raw sequencing reads and assembled genome sequences of strains AC027S^T^ (SRR35388761, GCA_056950985) and AC027N (SRR35388762, GCA_056951025) are available through the NCBI BioProject PRJNA1328261.

## Introduction

Leguminous plants establish a specialized symbiosis with nitrogen-fixing bacteria, mainly *Rhizobium* and related genera, whereby the bacteria colonize root nodules and convert atmospheric nitrogen into forms that the plant can use for growth [1,2]. This biological process not only provides plants with a direct nitrogen supply but also enriches soil nitrogen pools, making it a fundamental step of the nitrogen cycle [3,4]. However, root nodules are not exclusively inhabited by nitrogen-fixing rhizobia: a diverse range of non-nodulating bacteria has been reported in various leguminous plants [5,10], including trees adapted to arid and semi-arid environments such as *Acacia* [11,12] and *Prosopis* [13,14]. Since the composition of the nodule microbiome is strongly influenced by the microbial pool inhabiting the rhizosphere and surrounding soil [8,15,17], the occurrence of actinobacteria within these communities is expected [10,18], particularly in arid ecosystems, where they often dominate [19,20]. These soil-dwelling bacteria are not directly responsible for nodule organogenesis or nitrogen-fixing symbiosis in leguminous plants [21], but their metabolic versatility and ecological plasticity [22,24] enable them to influence microbial community dynamics within the nodules and contribute to plant resilience under climatic extremes [7,16, 25, 26]. They do so through multiple mechanisms, including the production of secondary metabolites and phytohormone-like compounds, modulation of plant immune responses and competition with potential pathogens [26,28]. However, members of the phylum *Actinomycetota* are not systematically detected in nitrogen-fixing nodules, and their presence is highly variable, differing between nodules and among host plants [26]. This variability is thought to reflect a combination of factors, including host genotype, soil characteristics and local environmental conditions, which together shape the composition and functional potential of the nodule microbiome.

In this study, we characterize two novel actinobacterial strains, AC027S^T^ and AC027N, isolated from globose and palmate root nodules collected from a 2-year-old grey-haired acacia planted in a desert site in Khurais, Saudi Arabia. Using a polyphasic taxonomic approach encompassing genotypic analysis and phenotypic characterization, we propose classifying these strains within the genus *Promicromonospora* and the establishment of a novel species. This genus, belonging to the family *Promicromonosporaceae* and order *Micrococcales*, comprises 14 species with validly published and correct names – according to the List of Prokaryotic Names with Standing in Nomenclature (LPSN) at the time of writing (11 March 2026). Its members have been isolated from diverse ecological niches, including soil, marine sediments, air and rhizosphere, as well as from several living hosts, such as plant root tissues and insects, but they have not yet been reported from root nodules [29].

## Methods

### Isolation and culture conditions

Grey-haired acacia (*Acacia gerrardii*=*Vachellia gerrardii*) in the *Fabaceae* (*Leguminosae*) family is a resilient, relatively fast-growing, semi-deciduous to deciduous tree well adapted to drought. It is commonly found in drylands across Africa and the Arabian Peninsula (https://www.gbif.org/species/3974764). During development, the root system of this leguminous plant establishes symbiotic relationships with several edaphic microorganisms, including nodule-forming bacteria [11,30]. The nodules used in this study were collected from plants uprooted 2 years after planting in the Khurais Desert, Saudi Arabia (25.260077° N 48.159163° E). During this period, the plants were irrigated daily with 2.5 l of water and fertilized every 3 months with NPK 20–20–20 (10 g per plant) supplemented with micronutrients (Fe-S-l-A, 215 mg per plant). Once transferred to the laboratory, nodules were visualized under a stereomicroscope and classified as globose and palmate (Fig. S1A and B, available in the online Supplementary Material). Intact nodules were further individually surface-sterilized with 70% ethanol for 2 min, followed by 1% sodium hypochlorite for 7 min, and then rinsed several times with sterile water. The surface-sterilized nodules were then separated from the main root with a sterile scalpel and transferred to a Petri dish, where they were smashed and homogenized in physiological solution (9 g l^−1^ NaCl). Serial dilutions were prepared and spread on plates of yeast extract mannitol agar, which was prepared as follows: yeast extract 0.4 g l^−1^, mannitol 10 g l^−1^, K_2_HPO_4_ 0.5 g l^−1^, MgSO_4_ 0.2 g l^−1^, NaCl 0.1 g l^−1^, agar 15 g l^−1^ and pH 6.8–7.0. Plates were incubated aerobically at 28 °C, and bacterial growth was checked daily to assess colony development. Based on their different morphologies (representative images in Fig. S1C), 50 colonies were picked from agar media and individually streaked onto tryptic soy agar (TSA) (Sigma-Aldrich) plates; the procedure was repeated to ensure the purification of bacterial culture. The obtained bacterial isolates were stored in 20% sterile glycerol at −80 °C. Routine cultivation of the isolates was performed at 28 °C on TSA plates under aerobic conditions.

### 16S rRNA gene sequencing and phylogeny

Genomic DNA was extracted from fresh cultures using a boiling lysis method and employed as template for PCR amplification of the 16S rRNA gene with standard primers 27F (5′-AGA GTT TGA TCM TGG CTC AG-3′) and 1492R (5′-GGT TAC CTT GTT ACG ACT T-3′) [31,32]. The resulting amplicons were sequenced at the Bioscience Core Laboratory (BCL), KAUST, Saudi Arabia. Following nucleotide trimming based on quality, forward and reverse reads were assembled into a single consensus sequence (~1,370 bp fragments). Comparison of the 16S rRNA gene sequences of the isolates with those available in the National Center for Biotechnology Information (NCBI) database (https://blast.ncbi.nlm.nih.gov/Blast.cgi) and the EzBioCloud database (https://www.ezbiocloud.net/identify) revealed that five isolates – three from the globose nodule (AC014S, AC026S and AC027S^T^) and two from the palmate nodule (AC025N and AC027N) – lacked conclusive matches to known type strains and were selected for further characterization. A phylogenetic tree based on the 16S rRNA gene sequence of the five isolates and related type strains within the *Promicromonospora* genus was constructed by using the neighbour-joining and maximum-likelihood methods in mega X (v10.1.8) with 1,000 resampling bootstraps after aligning the sequences with the sina software [33,34].

### Genome sequencing, assembly and annotation

Among the five isolates, we selected one per nodule type, resulting in the genome sequencing and analysis of AC027S^T^ and AC027N. Genomic DNA was extracted using the QIAGEN Genomic-tip 100 G^−1^ kit starting from bacterial cultures grown at 28 °C in TSA plates. The extracted DNA was quantified using the Qubit dsDNA High Sensitivity Assay Kit (Thermo-Fischer Scientific), and its quality was checked using a Bioanalyzer 2100 (Agilent) before being sequenced on an ONT GridION at BCL, KAUST. Whole-genome assemblies were obtained using the HGAP.3 workflow (v2.3.0) [35]. The contigs were assembled into scaffolds with the RagTag (v2.10) software [36]. The Bakta genome annotation pipeline (v1.8.2), eggNOG-mapper (v2.12) and microTrait software (v1.0.0) were used for genome annotations and metabolic pathways identification via the KEGG pathway database [37,40], while geNomad (v1.7) was used to determine the presence of plasmids and prophages in the genomes [41]. Putative secondary-metabolite biosynthetic gene clusters were predicted from the assembled genomes of strains AC027S^T^ and AC027N using antiSMASH v8.0.0 with default settings, enabling KnownClusterBlast, SubClusterBlast, ActiveSiteFinder, RREFinder and TFBS analysis [42]. Prediction of plant growth-promoting traits (PGPT) was performed using the PGPT-Pred tool on the PLaBAse web server [43,44]. Genomes of our strains were subsequently compared with all *Promicromonospora* species for which genome sequences are available among those with validly published names in LPSN (Table S1), using the pangenomic workflow implemented in anvi’o (version 8) [45].

### Phylogenomic analysis and comparative genomic analysis

A multi-locus sequence analysis was performed using a phylogenetic tree reconstructed from the alignment of 120 concatenated, single-copy, conserved marker genes from bacterial genomes. The analysis was conducted *de novo* using the GTDB-Tk software [46] on the genomes of strains AC027S^T^ and AC027N, along with 11 genomes of type strains from the genus *Promicromonospora* and 8 representatives of other genera within the family *Promicromonosporaceae*, including *Myceligenerans*, *Xylanimonas* and *Antribacter* (species names and accession numbers in Table S1 and completeness and contamination values in Table S2). The tree was rooted using *Micrococcus luteus* NCTC 2665^T^ as an outgroup. The final bootstrapped phylogeny was visualized with FigTree (v1.4.4) [47]. In addition, to account for whole-genome variation, we uploaded our genome sequences to the Type (Strain) Genome Server (TYGS), a free bioinformatics platform available at https://tygs.dsmz.de [48,50]. Briefly, the most closely related type strains were identified using a two-step approach that combined genome-wide similarity estimates via MASH searches [51] with 16S rRNA-based blast screening [52] against all type strains available in TYGS. Accurate intergenomic distances between the selected genomes were inferred using the algorithm ‘trimming’ and distance formula *d_5_* [53] and further used to infer a balanced minimum-evolution tree with branch support using FASTME 2.1.6.1 [54], including Subtree Pruning and Regrafting (SPR) post-processing. Branch support was inferred from 100 pseudo-bootstrap replicates each. The trees were rooted at the midpoint and visualized with PhyD3 [53].

Genome-wide relatedness between strains was also evaluated. Pairwise average nucleotide identity based on blast (ANIb) was calculated using JSpeciesWS with default parameters [55]. Microbial Species Identifier (MiSI) values were calculated using the genome-wide average nucleotide identity (gANI) and the alignment fraction (AF) [56]. Digital DNA–DNA hybridization (dDDH) values were determined *in silico* with the Genome-to-Genome Distance Calculator web server [57,58]. In addition, genome-relatedness indices at the protein level, including average amino acid identity (AAI) and percentage of conserved proteins (POCP), were computed with the EzAAI workflow and the Holzer POCP pipeline, respectively [59,62]. Genome quality (i.e. completeness and contamination) was determined with checkm2 (v1.1.0) for all strains analysed [63].

### Assessment of nodulation enhancement mediated by AC027S^T^ in desert soil

The potential of strain AC027S^T^ to support and/or promote nodulation by rhizobia naturally present in the desert soil of Khurais was tested using *A. gerrardii* plantlets. Briefly, *A. gerrardii seeds* were immersed in water at room temperature for 72 h to break physical dormancy. Swollen seeds were then transferred onto moistened filter paper placed in Petri dishes and incubated in the dark at 25–28 °C. Filter papers were kept consistently moist throughout the germination period. Germinated seedlings with radicles >1 cm were transferred to soil microcosms containing Khurais’ soil and maintained under controlled growth chamber conditions (16 h light/8 h dark, 25 °C and 50% relative humidity). Following a 1-week acclimation period, 20 growing plantlets were selected and equally allocated to either the control or AC027S^T^-inoculated treatment. The bacterium was grown on tryptic soy broth (TSB) at 28 °C, harvested, washed twice in sterile physiological saline solution and adjusted to a final concentration of 10^8^ cells per 100 g of desert soil. Control treatments consisted of uninoculated native soil. Nodulation was assessed after 45 days by gently uprooting the plants and examining the entire root system under a stereomicroscope. The number of nodules per plant was quantified, and differences between bacteria-inoculated and non-inoculated plants were statistically evaluated using ANOVA (significance, *P*<0.05).

### Morphological, physiological and biochemical characterization

Cell morphology was visualized via Zeiss Merlin scanning electron microscopy (SEM) at the Imaging and Characterization Core Lab at KAUST. ImageJ software was used to determine the dimensions of individual cells. Gram staining was performed following the standard protocol [64]. To evaluate cell motility, 10 µl of liquid culture was inoculated into soft agar (0.3% [w/v] agar) medium and incubated at 28 °C for 72–96 h (adapted from [65]). Spore formation was tested by exposing resuspended cells grown on TSA plates for 5 days to 80 °C for 10 min and then inoculating onto fresh plates and incubating at 28 °C to assess survival and regrowth, according to standard procedures. Growth under microaerophilic or anaerobic conditions was tested with CampyGen and AnaeroGen (Thermo Scientific), respectively. Bacteria were incubated at 10, 15, 20, 25, 28, 30, 37, 40, 42 and 50 °C for 7 days to determine temperature tolerance and the optimal temperature. Halotolerance and growth capacity at different pH levels were investigated using the Phenotype Microarray Biolog PM9 and PM10 plates, respectively, with TSB. Growth on single carbon sources was assessed by inoculating Phenotype Microarray Biolog PM1 and PM2 into the IF0 solution. The capacity to grow on Luria–Bertani (LB) agar, potato-dextrose agar (PDA), Reasoner’s 2A agar (R2A), nutrient agar (NA), rice extract agar and malt extract agar was also tested. Catalase activity was determined by adding a drop of 3% (v/v) H_2_O_2_ to whole bacterial cells and oxidase activity by adding a tetramethyl-p-phenylenediamine solution to a cell suspension. API 50CH B /E, API ZYM and API 20 NE test kits (bioMérieux) were used according to the manufacturer’s recommendations for the additional phenotypic tests. Other enzymatic activities, including cellulase, amylase, casein hydrolase, xylanase and pectinase, were tested using standard plate assay methods. Substrate hydrolysis was assessed by growth on agar media supplemented with the respective polymers, followed by visualization of clear zones around colonies after appropriate staining or indicator treatment [66].

The two isolates, AC027S^T^ and AC027N, were compared with five reference type strains. To capture the relevant genomic diversity within the genus *Promicromonospora* and to evaluate both fine-scale relatedness and broader phylogenomic structure, two type strains were selected: *Promicromonospora thailandica* S7F-02^T^ (DSM 26652) [67] and *Promicromonospora soli* NEAU-GS50^T^ (DSM 104515) [68]. In addition, three type strains representing other genera within the *Promicromonosporaceae* family, i.e. *Myceligenerans xiligouense* XLG9A10.2^T^ (DSM 15700) [69], *Xylanimonas cellulosilytica* XIL07^T^ (DSM 15894) [70] and *Antribacter gilvus* CFH 30434^T^ (KCTC 49093) [71], were included to place the novel isolates within a broader phylogenomic and taxonomic framework. This comparative approach strengthens the taxonomic interpretation and provides a robust assessment of the evolutionary distinctiveness and diversification of the newly proposed species, which is particularly relevant for *Actinomycetota*, where horizontal gene transfer can blur genus-level boundaries and produce overlapping physiological or chemotaxonomic traits [72,73]. All strains were cultivated under the same conditions used for AC027S^T^ and AC027N, as specified for each assay.

### Chemotaxonomic characterization

Analyses of respiratory quinones, polar lipids, cellular fatty acids and cell walls were conducted by the DSMZ services at the Leibniz-Institute DSMZ following the standard methodology. The chemotaxonomic characterization was conducted using cells collected from TSA plates incubated at 28 °C for 3 days. The five reference type strains selected (S7F-02^T^, NEAU-GS50^T^, XLG9A10.2^T^, XIL07^T^ and CFH 30434^T^) were cultivated under the same incubation conditions and analysed for comparative purposes.

## Results and discussion

### Identification and phylogenetic placement based on the 16S rRNA gene sequences

**Fig. 1.**
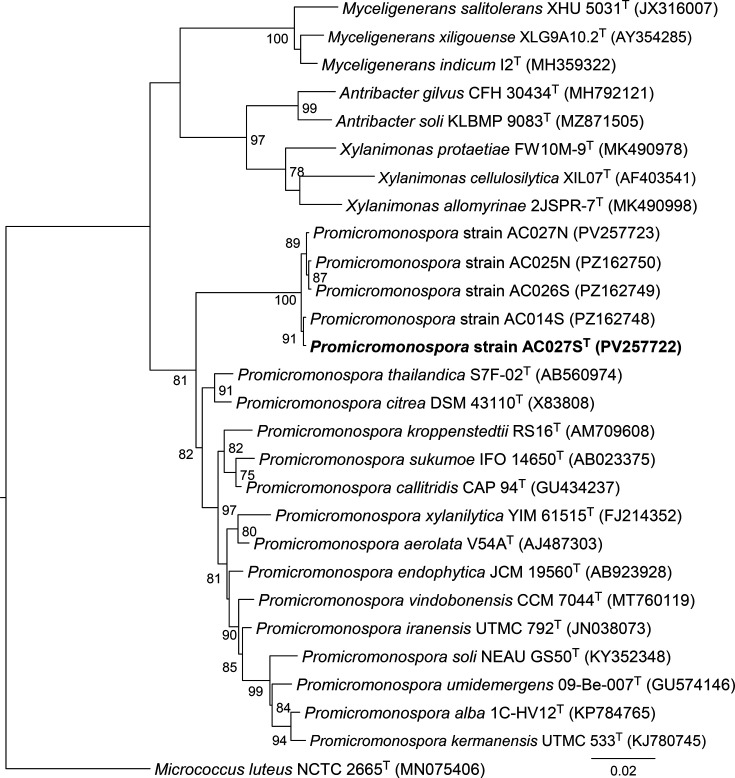
Phylogenetic tree based on 16S rRNA gene sequences from the five isolates (AC014S, AC026S, AC027S^T^, AC025N and AC027N) and closely related strains. The tree was reconstructed using the neighbour-joining method in mega X. Filled circles indicate branches that were also recovered using the maximum-likelihood method. Numbers at the branching points indicate bootstrap values (expressed as percentages of 1,000 replications), and only those >50% are shown. *Micrococcus luteus* NCTC 2665^T^ (MN075406) was used as an outgroup. Bar, 0.02 substitutions per nucleotide position.

The five isolates that could not be unambiguously assigned to any recognized type species showed the highest 16S rRNA gene sequence similarity to *P. thailandica* S7F-02^T^, with values ranging from 96.49 to 97.42%. Pairwise 16S rRNA gene sequence similarities among the isolates were all above 99.5% (Fig. S2). In the 16S rRNA gene maximum-likelihood phylogeny, the five isolates clustered together within the genus *Promicromonospora*, forming a distinct and well-supported external branch relative to the other type strains, with *P. thailandica* S7F-02^T^ and *Promicromonospora citrea* DSM 43110^T^ as the closest related cluster (Fig. 1). Although the 16S rRNA gene sequence identities to the closest related strains were above the generally accepted threshold for genus delineation (<95%) [74], the observed phylogenetic separation suggests that these strains may constitute a distinct evolutionary lineage within the genus. This branching pattern may reflect long-term divergence associated with adaptation to the nodule microenvironment, pointing to the potential emergence of a distinct genus. Considering the ecology of the species with validly published names currently assigned to the genus *Promicromonospora*, none has been isolated from leguminous hosts or nitrogen-fixing root nodules. Described members of the genus have predominantly been isolated from arid or semi-arid environments, either in association with plant tissues (*P. citrea* DSM 43110^T^, *Promicromonospora callitridis* CAP 94^T^, *Promicromonospora endophytica* JCM 19560^T^ and *Promicromonospora xylanilytica* YIM 61515^T^) or from soil (*Promicromonospora iranensis* UTMC 792^T^, *Promicromonospora kermanensis* UTMC 533^T^, *Promicromonospora kroppenstedtii* RS16^T^ and *P. soli* NEAU GS50^T^). The isolation of strains AC027S^T^ and AC027N from *Acacia* root nodules, therefore, expands the known ecological range of the genus and suggests a previously unrecognized association with plant–microbe symbioses, specifically within nitrogen-fixing nodules.

### Phylogenomic and comparative genomic analysis

**Fig. 2.**
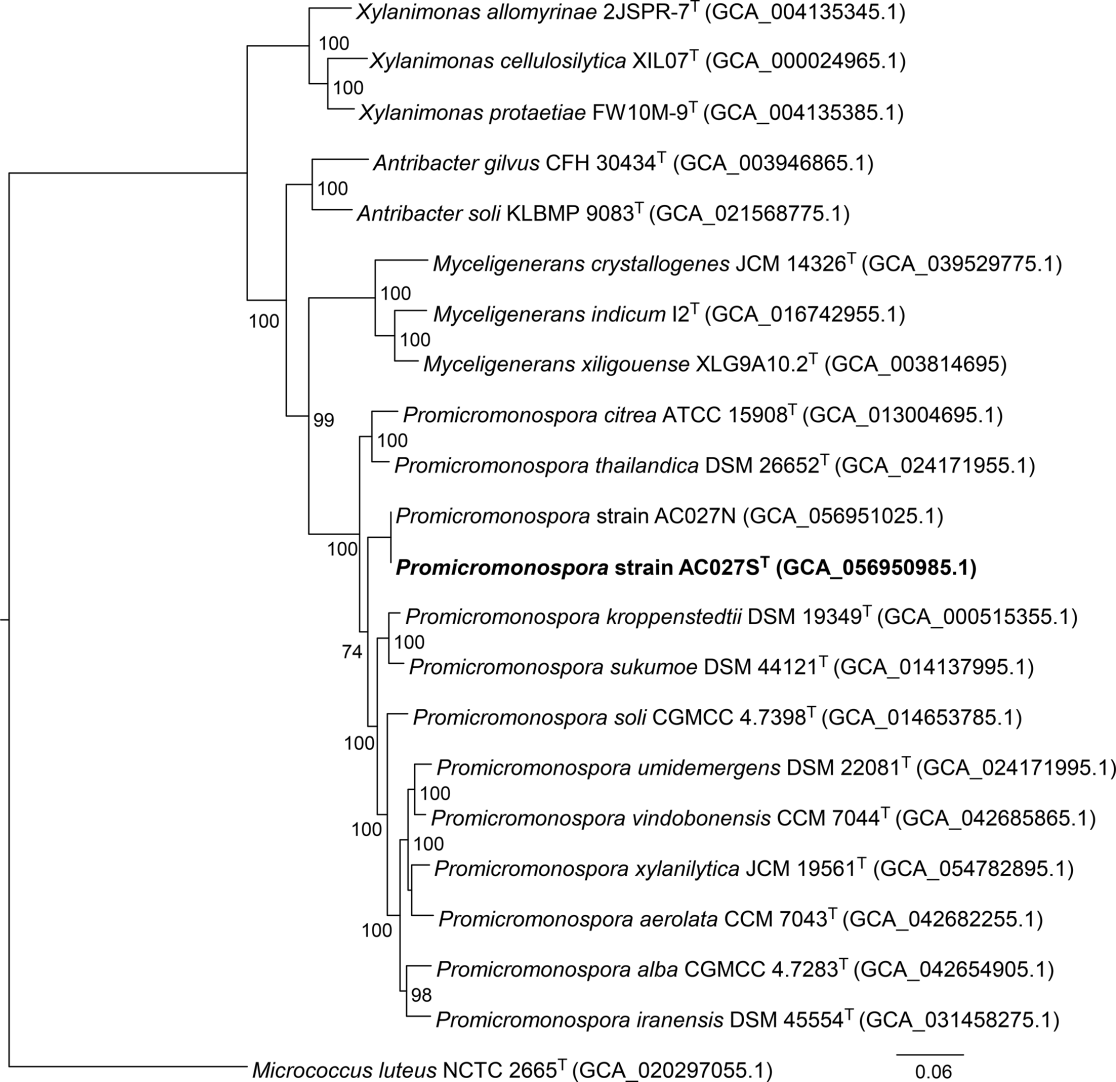
Phylogenomic tree of *Promicromonospora* strains AC027S^T^ and AC027N together with related genera, including *Xylanimonas*, *Antribacter* and *Myceligenerans*. The tree was constructed using the maximum-likelihood method on 120 concatenated single-copy genes with GTDB-Tk [46] and rooted with *Micrococcus luteus *NCTC 2665^T^. NCBI accession numbers are reported after the species name. The tree is drawn to scale, with branch lengths measured in substitutions per site. Numbers at nodes indicate bootstrap percentages using 1,000 replicates. Bar, 0.06 substitutions per site. Completeness and contamination values for the genomes used in the tree are shown in Table 2 and Table S2.

The alignment of the 120 concatenated genes showed that the two selected strains, AC027S^T^ and AC027N, form an independent clade distinct from other *Promicromonospora* species (Fig. 2). Over the 120 core proteins, the branch length distances to the AC027S^T^ and AC027N tips are 0.055, 0.059 and 0.06 substitutions per amino acid for *P. kroppenstedtii* RS16^T^, *Promicromonospora sukumoe* IFO 14650^T^ and *P. thailandica* S7F-02^T^, respectively, corresponding to a 5.5–6% divergence across highly conserved housekeeping proteins. The whole-genome-based phylogenomic analysis confirmed that our isolates form a distinct clade within the genus *Promicromonospora* (Genome BLAST Distance Phylogeny [GBDP] tree in Fig. S3), although the inferred topology identified *P. soli* NEAU-GS50^T^ as their closest described relative. The evolutionary distances observed in both phylogenomic analyses thus support the placement of AC027S^T^ and AC027N as a distinct lineage within *Promicromonospora* that represents an early-diverging branch of the genus, thereby expanding the genus’s phylogenetic breadth.

Nucleotide-level similarity across orthologous genome regions further confirmed that AC027S^T^ and AC027N represent the same species (ANIb and dDDH=100%, Table 1). When compared to the type strains of the genus *Promicromonospora,* instead, both ANIb and dDDH showed values below the established thresholds for species delineation, i.e. 95–96% for ANIb and 70% for dDDH [55,58, 75,78]. Specifically, ANIb ranged from 85.18 to 86.76%, whereas dDDH values ranged from 27.4 to 31.9% (Table 1), with *P. soli* NEAU-GS50^T^ showing the highest similarity across shared genome sequences for both genomic metrics [55,58, 75,78]. Similarly, according to the MiSI thresholds (gANI ≥96.5% and AF ≥60%) [56], strains were considered distinct from known species (Table 1). At the protein level, all comparisons of protein-coding genes (orthologous proteins, i.e. indicated by AAI) and of proteins shared between two genomes (i.e. POCP) had values above the proposed genus-level cut-offs (Table 1) [61,76], consistent with the assignment of strains AC027S^T^ and AC027N to a novel species within the genus *Promicromonospora*. However, while genomic indices clearly place them within this genus, their distinct phylogenetic position and unique occurrence in legume nodules in arid soils highlight the potential for long-term evolutionary divergence.

**Table 1.** ANIb, AF, *in silico* dDDH, MiSI combining gANI with AF, AAI and POCP of AC027S^T^ compared with AC027N (this study) and available genomes from ten type strains of the genus *Promicromonospora* (Table S1) All values are expressed as a percentage (%). The commonly accepted thresholds for species delineation are ANIb/gANI ≥95%, AF≥ 60% and dDDH ≥70% [55,75], whereas the genus-level delineation is generally defined by an AAI of ~65% and a POCP of ~50% [61,76].

Genomic metrics for AC027S^T^	ANIb	AF	MiSI	dDDH	AAI	POCP
AC027N	100.00	99.99	99.99	100.00	100.00	99.80
*P. soli* NEAU-GS50^T^	86.07	54.11	46.57	31.90	85.20	70.69
*P. kroppenstedtii* RS16^T^	84.99	54.64	46.44	29.80	84.23	75.01
*P. sukumoe* IFO 14650^T^	84.79	51.56	43.72	29.80	83.53	73.19
*P. iranensis* HM 792^T^	84.69	52.76	44.68	29.60	83.54	72.37
*P. vindobonensis* V-45^T^	84.55	50.53	42.72	29.30	83.36	72.71
*P. alba* 1C-HV12^T^	84.53	49.69	42.01	29.70	83.32	73.15
*P. umidemergens* 09-Be-007^T^	84.52	47.42	40.08	29.20	83.07	69.81
*P. aerolata* V-54A^T^	84.42	52.04	43.93	29.10	83.33	72.68
*P. thailandica* S7F-02^T^	84.02	57.04	47.93	28.50	83.11	74.50
*P. xylanilytica* JCM 19561^T^	83.81	56.38	47.98	29.10	83.60	73.43
*P. citrea* LL G-165^T^	83.07	57.73	47.96	27.00	82.01	73.86

### Genome features, adaptation to nodule niches and support for nodulation efficiency

The genomes of AC027S^T^ and AC027N measured 6.03 and 6.04 Mb, respectively, with a G+C content of 72.1%, completeness of 99.97% and contamination of 1% (Table 2). Each genome assembly consisted of a single contig, comprising 5,287 and 5,303 predicted genes, of which 5,204 and 5,220 were protein-coding sequences. In comparison with the genomes of the ten reference *Promicromonospora* type species (details in Table S1), the size of AC027S^T^ and AC027N was close to the genus median (median=6.1 Mb, first quartile=5.45 Mb and third quartile=6.62 Mb). Key genomic features of AC027S^T^ and AC027N include the presence of 9 rRNA genes (three 5S, three 16S and three 23S rRNA genes), 51 copies of tRNA and 2 integrated prophages (Table 2). For the 16S rRNA gene specifically, three copies were retrieved for each genome, all showing 100% sequencing identity to one another and more than 99.5% identity to the sequences obtained by PCR amplification (Fig. S4).

**Table 2.** Genome characteristics of the strains AC027S^T^ and AC027N obtained in this study, together with those of two type strains of the genus *Promicromonospora* and representative type species of three closely related genera within the family *Promicromonosporaceae* Strains: 1, AC027S^T^ (this work, SAMN51309524); 2, AC027N (this work, SAMN51309523); 3, *P. thailandica* S7F-02^T^ (GCA_024171955); 4, *P. soli* NEAU-GS50^T^ (GCA_014653785); 5, *M. xiligouense* XLG9A10.2^T^ (GCA_003814695); 6, *X. cellulosilytica* XIL07^T^ (GCA_000024965); 7, *Antribacter gilvus* CFH 30434^T^ (GCA_003946865). *Determined using geNomad [41].

Characteristic	1	2	3	4	5	6	7
Size (bp)	6,027,935	6,039,039	5,365,806	5,371,896	4,723,192	3,831,380	5,149,130
Contigs	1	1	37	16	1	2	30
DNA G+C (%)	72.1	72.1	73.1	71.7	71.7	72.5	4,697
Coding sequence	5,208	5,222	4,889	4,803	4,042	3,464	1,857
Plasmid*	0	0	0	1	0	1	7
Prophages*	0	0	0	1	0	0	0
No. of rRNA genes							
5S	3	3	0	3	3	3	1
16S	3	3	1	1	3	3	2
23S	3	3	1	1	3	3	2
No. of tRNA	51	51	51	51	54	51	54
Completeness (%)	99.97	99.97	99.94	100	99.98	99.94	100
Contamination (%)	1.00	1.02	0.47	0.43	0.04	0.42	0.53

Functional genomic analyses showed that AC027S^T^ and AC027N strains are mixotrophs capable of both aerobic and anaerobic respiration, possessing a complete electron transport chain with complexes II–IV for oxidative phosphorylation. They encode enzymes required for performing denitrification (*narGH*, AC0QU2_02263-64) and nitrite oxidation (*nirBD*, AC0QU2_00642-43) through chemolithoautotrophic pathways. The strains lack mobility genes governing chemotaxis, flagella or pili but possess genes involved in quorum sensing. In terms of substrate utilization, the strains possess a vast repertoire of transport systems for the uptake of sugars (mono-, di-, oligo- and poly-saccharides), sugar alcohols, amino acids, lipids, nucleotides, vitamins, peptides, metal ions and osmolytes. They also carry the genetic potential to degrade a broad range of carbon substrates, from simple sugars (e.g. fructose, maltose, mannose and trehalose) to complex plant-derived polymers, such as cellulose, pectin, chitin, xylan, xyloglucan and heteromannan, which may facilitate bacteria to breach plant cell walls, access nutrients and establish intimate contact with host tissues, such as the root nodules. As well, strains AC027S^T^ and AC027N harboured three of the seven genes typically involved in exopolysaccharide (EPS) biosynthesis (*epsC*, *epsD* and *epsP*, i.e. AC0QU2_03939, AC0QU2_01685, AC0QU2_04358, respectively), indicating a genetic capacity to assemble or export partial exopolysaccharide structures or surface glycoconjugates that may support adhesion, colonization or biofilm formation rather than standard EPS production.

From an adaptive perspective, the two strains encode genes involved in stress responses, including those that respond to salinity, temperature and oxygen levels. In response to heat stress, the bacterial strains could produce heat shock proteins and ATP-dependent proteases to refold or degrade misfolded proteins, with heat-induced transcription factors regulating these stress-responsive genes. Additionally, Reactive Oxygen Species (ROS) scavenging enzymes and biosynthetic genes for compatible solutes, such as ectoine (*ectC*, AC0QU2_02699) and proline (*proC*, AC0QU2_00801), can help the strains cope with oxidative and osmotic stress.

Analysis of plant-associated metabolic pathways (biopromotion and biofertilization) revealed that strains AC027S^T^ and AC027N possess the complete tryptophan biosynthesis pathway and genes for the production of auxin precursors, as well as several genes of the indole-3-pyruvate auxin biosynthesis pathway, although not the whole set. These intermediates, however, could be metabolized by other members of the nodule microbiome to produce indole-3-acetic acid, highlighting the potential for a metabolic continuum within the community. We also detected two variants of the *nodU* gene (AC0QU2_02541, AC0QU2_00836), which encodes a carbamoyltransferase involved in nodule function. In classical rhizobia, *nodU* is involved in the carbamoylation of Nod factors, influencing nodule development and host specificity. However, in non-rhizobial bacteria, *nodU* homologues have also been linked to broader adaptive roles, such as modifying extracellular molecules and mediating interactions within the nodule microbiome, as part of a ‘rhizobia-helper’ adaptive, functional strategy [79]. Likewise, the detection of *cysN* (ATP sulfurylase large subunit, AC0QU2_04680) and *cysC* (APS kinase, AC0QU2_02244) as part of the assimilatory sulphate reduction pathway highlights the capacity of our strains to potentially convert inorganic sulphate (SO_4_^2-^) into reduced sulphur compounds used in amino acids (cysteine and methionine) and cofactors [80]. Cysteine, in particular, serves as a precursor for glutathione and homoglutathione, which act as redox buffers and protect nodule cells from oxidative stress. A sulphur deficiency can thus disrupt nitrogenase activity, impair leghemoglobin function and compromise nodule metabolism, ultimately reducing nitrogen fixation efficiency [81]. The genomes of AC027S^T^ and AC027N also encoded the *ModABC* high-affinity molybdenum transport system (AC0QU2_03115–17), TonB-dependent uptake systems for siderophores and genes for siderophore biosynthesis (AC0QU2_02673–79), including a cluster predicted to produce a non-iron-chelating siderophore. Together, genes encoding iron-storage proteins, such as ferritin (*bfr,* AC0QU2_02592), were found, which may enable these new actinobacteria to concentrate essential metals that serve as cofactors for the rhizobial nitrogenase. Yet, the detection of operons involved in niacin (vitamin B3) biosynthesis (*nadABCD*, AC0QU2_01945, AC0QU2_05097, AC0QU2_05094, AC0QU2_04309) [82] suggests that our strain may act as a metabolic helper by improving metabolic efficiency, energy production and stress tolerance.

Upon examining the pangenome of *Promicromonospora*, which included the genomes of strains AC027S^T^ and AC027N together with those of the available type species [*n*=11, Table S1; core genome size=2,444 gene clusters (19%); accessory genome=5,975 gene clusters (47%)], we identified that the unique gene complement of our strains was composed of 740 gene clusters, i.e. 6% of all gene clusters and 15% of our strains gene clusters. The AC027S^T^ and AC027N-specific gene clusters were primarily associated with functions involved in siderophore transport and biosynthesis, nickel resistance, degradation of xenobiotic compounds such as benzoate and dichloroethane and degradation and transport of diverse polysaccharides (e.g. chitin, cellulose, galactose, fructose and glucose). Notably, genes associated with antibiotic resistance were also present uniquely in AC027S^T^ and AC027N (*vanRYW*, AC0QU2_01518, AC0QU2_01771, AC0QU2_04549), distinguishing them from other members of the genus *Promicromonospora*. The enrichment of these functions suggests that the novel strains are adapted to withstand metal stress, exploit plant- and microbe-derived polymers and compete effectively within the microbial consortia of legume nodules.

**Fig. 3.**
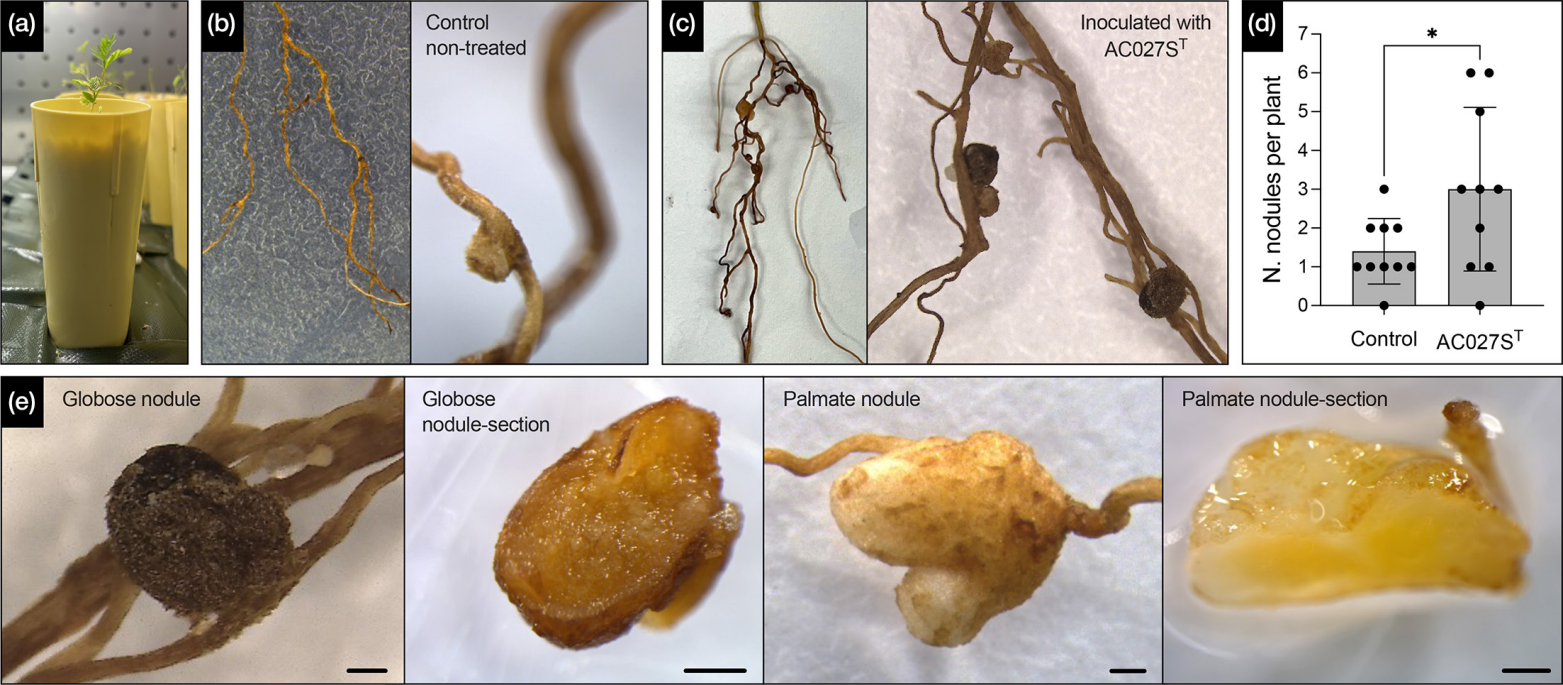
Nodulation assay of *A. gerrardii* plantlets grown in Khurais desert soil with or without inoculation with strain AC027S^T^. (**a**) Representative microcosms in which plantlets of acacia were transferred. (**b**) Control plants grown in desert soil without any inoculation, with an example of a root nodule developed after 45 days. (**c**) Representative root system of a plant inoculated with AC027S^T^ that had the highest number of nodules observed, along with magnification of developed nodules. (**d**) Bar plots (mean, sd) combined with values of nodules per plant measured in non-treated control plants (mean=1, sd=0.8, min=0 and max=3, *n*=10) and plants inoculated with AC027S^T^ (mean=3, sd=2, min=0 and max=6, *n*=10). Start indicates the statistical difference between the two groups (Welch–corrected *t*: t=2.228, df=11.81, *P*=0.0461). (**e**) Representative images of globose and palmate nodules from the root systems of plants inoculated with AC027S^T^ and their cross-dissection. Bar length is 1 mm.

To assess whether the metabolic and ecological traits of the new species could support nodulation in *A. gerrardii*, a microcosm assay was conducted using the original Khurais desert soil, which hosts nodule-forming bacteria, as observed in the original adult plants uprooted and sampled. In the control group, most plantlets (9/10) developed a few nodules, limited to 1–3 per plant. In contrast, plants inoculated with strain AC027S^T^ formed up to six nodules per plant, which were visibly larger than those in the control (Fig. 3). These results suggest that while AC027S^T^ is not expected to induce nodulation independently (see genome analysis), it can enhance nodulation when native nodulating bacteria are present in the soil.

### Cell morphology and colony characteristics

**Fig. 4.**
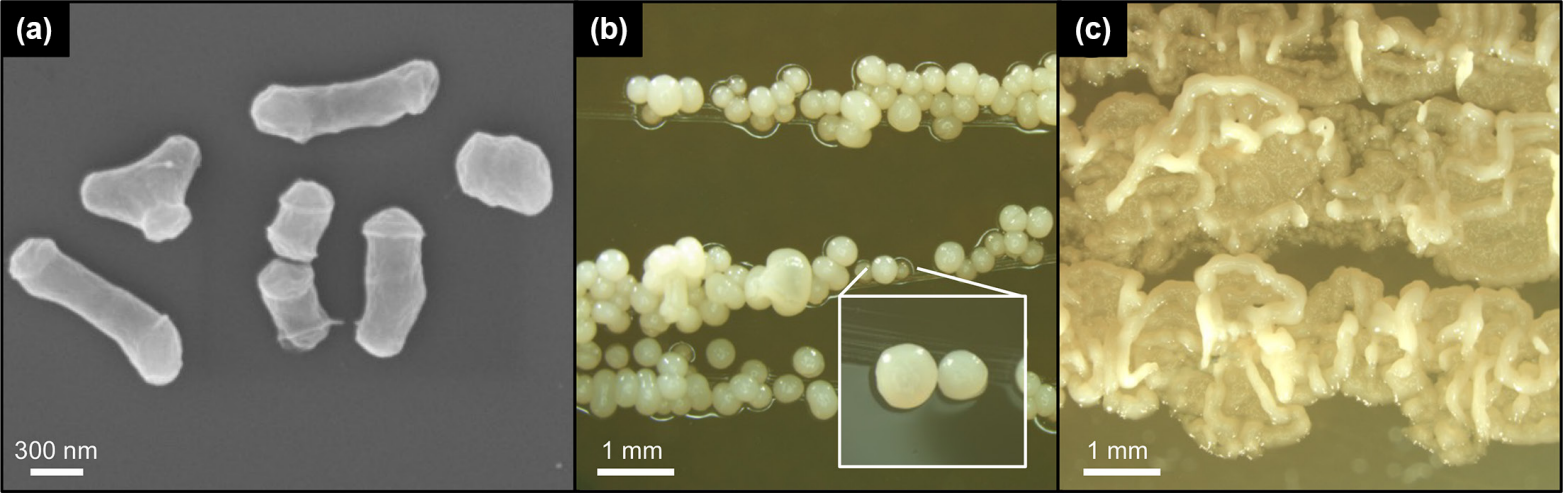
Cell and colony morphology of *Promicromonospora* strain AC027^T^. (**a**) SEM micrograph of AC027S^T^ cells grown on TSB medium at 28 °C showing irregular rod shapes at nanometre scale. Bar length is 300 nm. Cell images at different magnifications are reported in Fig. S5 for both AC027S^T^ and AC027N strains. Optical images of AC027S^T^ colonies on TSA plates at (b) n error in days and (c) 7 days revealing spherical smooth colonies that develop into larger wrinkled structures. Bar length is 1 mm.

At SEM, the cells of strains AC027S^T^ and AC027N were rugose, irregular and occurred as short rods to coccoid-like forms that were arranged singly, in pairs or occasionally in short chains (Figs 4a and S5). Cell dimensions ranged from 0.4 to 1.1 µm in length, with a homogeneous width of 0.25±0.06 µm. They were Gram-stain-positive, non-sporulating and non-motile. When grown on TSA medium at 28 °C for 3 days, colonies were creamy beige, circular, with regular margins, and reached an average diameter of 0.4 mm (Fig. 4b). After extended incubation (7 days) or under confluent growth, colonies developed a roughened surface with a complex three-dimensional structure (Fig. 4c), in which branched filaments extended farther into the agar, often forming interwoven structures. Such growth features are consistent with those reported for other members of the genus *Promicromonospora* and, more generally, for other actinobacteria that grow on solid surfaces.

### Growth preferences and biochemical reactions

The strains AC027S^T^ and AC027N were mesophilic (optimum 28–30 °C) with an extended growth range from 20 °C to moderate thermophilic conditions (42 °C), slightly halotolerant [0–3% (w/v) NaCl, optimum 0–1%] and grow over a wide pH range (5–10.0, optimum 7–8%) (Table 3). They were aerobic but can grow under microaerobic conditions. AC027S^T^ and AC027N can also grow on LB, R2A, NA and PDA plates, but they did not grow on rice extract and malt extract plates. The two endophytic actinobacteria residing in plant nodules are well-adapted to utilize a diverse array of carbon sources derived from plant host tissues and those metabolites produced by their metabolic activity [83]. Specifically, strains AC027S^T^ and AC027N actively metabolized a range of compounds as sole carbon sources. These included simple sugars, such as arabinose, xylose, ribose, lyxose, maltose and glucosamine – typically derived from cell wall remodelling – and more complex polysaccharides, such as dextrin, gentiobiose, glycogen and pectin, which are components of cell walls and storage tissues. Additionally, they utilized dicarboxylic acids, such as malate, succinate and fumarate, which are commonly secreted by plants. These metabolic capabilities align with genomic features and underscore the strains’ adaptation to the nodule environment. The two strains were also able to hydrolyse cellulose, starch and casein (Table 4), which may provide access to diverse nutrient sources in the rhizosphere and nodules, thereby supporting adaptation to the plant environment and facilitating colonization [84,85]. AC027S^T^ and AC027N have catalase-positive, oxidase-positive and peroxidase-negative phenotypes. Nitrate reduction was weak (compared to *Escherichia coli*), and indole production was negative. According to the API 20NE test strip, the strains were positive for aesculin and gelatine hydrolysis, *β*-glucosidase (using PNPG) and denitrification (reduction of nitrate to nitrogen gas), while weak reduction of nitrate to nitrite was confirmed. The API ZYM test strip shows positive activity for alkaline phosphatase, esterase (C4), esterase lipase (C8), leucine arylamidase, trypsin, acid phosphatase, naphthol-AS-BI-phosphohydrolase, galactosidase (*α* and *β*), glucosidase (*α* and *β*), *N*-acetyl-*β*-glucosaminidase and *α*-mannosidase; valine and cystine arylamidases showed both weak activities. The AC027S^T^ and AC027N strains ferment several carbohydrates (*n*=22), including, among others, erythritol, d-arabinose, d-ribose, methyl-*β*-d-xylopyranoside, d-galactose, d-glucose, d-fructose, aesculin, salicin, d-cellobiose, d-maltose, d-lactose (bovine origin), d-melibiose, sucrose (sucrose), starch and glycogen (API 50CH B/E test strips in Fig. S6). Notably, whereas the two *Promicromonospora* type species tested fermented only a limited number of substrates, strains AC027ST and AC027N displayed a broader profile, similar to that of *Antribacter gilvus* CFH 30434^T^ and *X. cellulosilytica* XIL07^T^.

**Table 3.** Morphological traits and growth preferences distinguishing strains AC027S^T^ and AC027N from the selected type strains Strains: 1, AC027S^T^; 2, AC027N; 3, *P. thailandica* S7F-02^T^; 4, *P. soli* NEAU-GS50^T^; 5, *M. xiligouense* XLG9A10.2^T^; 6, *X. cellulosilytica* XIL07^T^; 7, *Antribacter gilvus* CFH 30434^T^. Data were obtained in this study. +, Positive reaction; –, negative reaction; w, weak reaction.

Physiology and growth preference	1	2	3	4	5	6	7
Colony colour on TSA	Ivory opaque	Ivory opaque	Yellow opaque	Creamy opaque	Yellow opaque	Beige shiny	Yellow shiny
Strick appearance/texture	Rough	Rough	Rough	Crusty	Rough	Smooth	Smooth
Motility	–	–	–	–	–	–	–
Temperature range (°C)	20–42(w)	20–42(w)	10(w)–37	10(w)–30	10(w)–37	10–40	10(w)–37
Temperature optimum (°C)	28–30	28–30	28–30	28–30	28–30	28–30	28–30
pH range	5–10	5–10	7–10	5–10	4–10		6–10
pH optimum	7–9	7–9	8–9	7–10	7–9	7–8	8–10
NaCl range (%, w/v)	0–3	0–3	0–4	0–3	0–15	0–4	0–2
NaCl optimum (%, w/v)	0–1	0–1	0–1	0–1	1–7	0–1	0–1
Growth on							
LB	w	w	w	+	+	+	+
PDA	w	w	+	–	–	–	–
R2A	+	+	+	+	+	+	w
TSA microaerophilic	+	+	+	+	+	w	–
TSA anaerobic	–	–	–	–	–	–	–

**Table 4.** Biochemical characteristics distinguishing strains AC027S^T^ and AC027N from selected type strains Strains: 1, AC027S^T^; 2, AC027N; 3, *P. thailandica* S7F-02^T^; 4, *P. soli* NEAU-GS50^T^; 5, *M. xiligouense* XLG9A10.2^T^; 6, *X. cellulosilytica* XIL07^T^; 7, *Antribacter gilvus* CFH 30434^T^. Data were obtained in this study. +, Positive reaction; –, negative reaction; w, weak reaction.

Physiology	1	2	3	4	5	6	7
Catalase activity	+	+	+	+	+	–	w
Oxidase activity	+	+	–	–	–	+	–
Peroxidase activity	–	–	+	+	–	–	–
Nitrate reduction	w	w	–	–	–	+	w
Cellulase	w	w	+	–	–	–	–
Amylase	++	++	+	+	+	+	+
Caseinase	+	+	–	+	–	–	–
API 20NE, enzymatic activity							
Nitrate reduction/N_2_ gas (NO_3_)	w/+	w/+	–/–	–/–	–/–	+/+	+/+
Fermentation/oxidation (GLU)	–	–	–	–	–	w	–
Aesculin hydrolysis (ESC)	+	+	+	+	+	+	+
Gelatin hydrolysis (GEL)	+	+	+	+	+	–	+
*β*-Glucosidase (PNG)	+	+	+	+	+	+	+
API ZYM, enzymatic activity:							
Alkaline phosphatase	+	+	w	–	–	–	–
Esterase (C4)	+	+	w	+	–	+	+
Esterase lipase (C8)	+	+	+	+	+	+	+
Leucin arylamidase	+	+	w	+	+	+	+
Valine arylamidase	w	w	–	w	–	w	–
Cystine arylamidase	w	w	–	–	–	–	–
Trypsin	+	+	w	+	+	–	–
Acid phosphatase	+	+	–	w	–	w	–
NAP-phosphohydrolase	+	+	+	+	+	+	+
*α*-Galactosidase	+	+	–	+	w	w	w
*β*-Galactosidase	+	+	w	+	–	–	+
*β*-Glucuronidase	–	–	–	+	–	–	–
*⍺*-glucosidase	+	+	+	+	+	+	+
*β*-glucosidase	+	+	+	+	+	–	+
*N*-acetyl-*β*-glucosaminidase	+	+	–	+	–	+	+
*α*-Mannosidase	+	+	+	+	–	–	–
API 50CH B/E, acid production from							
Glycerol	+	+	+	+			
d-Xylose	+	+	+	+			
d-Glucose	+	+	+	+			
d-Mannose	+	+	+	–			
Inositol	+	+	w	–			
Aesculin	+	–	+	–			
Lyxose	+	–	w	–			
5-Keto-gluconate	+	+	–	w			

Detailed phenotypic, physiological and biochemical characteristics of the proposed strains and related type species are summarized in Tables 3 and 4.

### Chemotaxonomic properties

The respiratory quinones are all menaquinones (MK), with MK9 (H_4_) as the primary component in all the analysed strains, followed by MK9 (H_6_) and MK9 (H_2_) in our strains and (MK-8 H_4_) in the other strains (Table S3). The cellular fatty acid profiles showed distinct features for genus differentiation, with similarities between species (Table 5, Fig. S7). For instance, the predominant fatty acids in AC027S^T^ and AC027N were the saturated branched-chain acids iso-C_15:0_ (48.6% and 46.3%) and anteiso-C1_5:0_ (40.9% and 41.9%), a pattern also observed in *P. thailandica* S7F-02ᵀ and *P. soli* NEAU-GS50ᵀ. However, a higher proportion of iso-C_15:0_ and lower levels of anteiso-C_15:0_ were observed in AC027S^T^ and AC027N compared to the other *Promicromonospora* strains. Notably, the predominance of these branched-chain fatty acids in actinobacteria is often linked to regulation of membrane fluidity and adaptation to challenging environmental conditions, such as desiccation, salinity and temperature fluctuations [86]. C_15:0_ and C_16:0_ were present in AC027S^T^ and AC027N at trace levels (<1%), whereas they reached nearly 3% each in *P. thailandica* S7F-02^T^. Additionally, the unsaturated fatty acid anteiso-C_15:1_* ω1*0c was not detected in AC027S^T^ and AC027N, but it was present across all the other strains except *Antribacter gilvus* CFH 30434^T^. The polar lipid profile of AC027S^T^ and AC027N was characterized by diphosphatidylglycerol as the dominant component, followed by three unidentified glycolipids, one unidentified phosphatidylglycerol, four glycophospholipids, four and two unidentified phospholipids and four and three unidentified lipids (Fig. S8). Although their polar lipid composition was similar to that of the other type strains across different genera, the distribution and intensity of the majority of detected lipids differed notably. Specifically, *Promicromonospora* strains displayed more intense phosphatidylglycerol spots, whereas *P. thailandica* S7F-02ᵀ totally lacked lipids, and *P. soli* NEAU-GS50ᵀ only had one that differed from those of AC027S^T^ and AC027N. Yet, the polar lipid profiles of AC027S^T^ and AC027N were more complex, with 17 and 14 distinct polar lipid spots, respectively, compared to 10 in *P. soli* NEAU-GS50ᵀ and 9 in *P. thailandica* S7F-02ᵀ (Fig. S8). These differences suggest a unique chemotaxonomic signature, further distinguishing AC027S^T^ and AC027N from related taxa within the *Promicromonospora* genus.

**Table 5.** Cellular fatty acid contents of proposed strains AC027S^T^ and AC027N, together with reference type strains used in this study Strains: 1, AC027S^T^; 2, AC027N; 3, *P. thailandica* S7F-02^T^; 4, *P. soli* NEAU-GS50^T^; 5, *M. xiligouense* XLG9A10.2^T^; 6, *X. cellulosilytica* XIL07^T^; 7, *Antribacter gilvus* CFH 30434^T^. All data were obtained in this study (Fig. S7). The prevalent fatty acids (>10%) in strains AC027S^T^ and AC027N are highlighted in bold. Fatty acids present at <1% in all strains are not reported. tr, trace amount (<1.0 %); –, not detected.

Fatty acid		1	2	3	4	5	6	7
Straight-chain	C_14:0_	tr	tr	2.9	tr	–	9.4	tr
	C_15:0_	tr	tr	tr	–	tr	4.2	tr
	C_16:0_	tr	tr	2.7	tr	1.9	3.7	tr
Branched-chain	isoC_14:0_	1.0	1.0	1.2	tr	tr	4.8	12.6
	**isoC_15:0_**	**48.6**	**46.3**	**41.3**	**43.6**	**28.2**	**10.0**	**13.1**
	**anteisoC_15:0_**	**40.9**	**41.9**	**42.8**	**44.4**	**42.4**	**60.6**	**64.2**
	isoC_16:0_	3.7	4.2	2.4	2.1	5.5	4.1	4.6
	isoC_17:0_	1.4	1.4	TR	2.0	2.7		TR
	anteisoC_17:0_	2.8	3.2	3.9	4.3	11.1	1.5	1
Unsaturated	isoC_15:1_* ω*10c	tr	tr	tr	tr	3.5	–	tr
Fatty acids	anteisoC_15:1_* ω*10c	–	–	tr	1.0	3.0	–	1.6

## Protologue and description of *Promicromonospora noduliphila* sp. nov.

*Promicromonospora noduliphila* (no.du.li’phi.la. N.L. masc. n. *nodulus*, a small knot, nodule; Gr. masc. adj. *philos*, loving; N.L. fem. adj. *noduliphila*, nodule-loving, referring to the isolation from root nodules of *Acacia gerrardii* and the potential to stimulate nodulation).

Cells are Gram-stain-positive, aerobic, non-motile, non-spore-forming and occur as short rods to coccoid-like forms, singly, in pairs or occasionally in short chains. After 3 days at 28 °C, colonies on TSA are creamy beige, circular, with entire margins and ~0.4 mm in diameter. Extended incubation produces colonies with a rough, three-dimensional surface. Growth occurs at 20–42 °C (optimum 28–30 °C), at pH 5.0–10.0 (optimum 7.0–9.0) and with 0–3.0% (w/v) NaCl. The predominant menaquinone is MK-9(H4). The major cellular fatty acids are iso-C_15:0_ and anteiso-C_15:0_. The polar lipid profile contains diphosphatidylglycerol, phosphatidylglycerol and several unidentified glycophospholipids, glycolipids, phospholipids and lipids. Whole-cell hydrolysates contain glucose as the major sugar. Peptidoglycan contains muramic acid with alanine, glutamic acid and lysine. It is positive to catalase and oxidase, but negative to peroxidase. Nitrate reduction is weak, and indole production is negative. The strain is positive for aesculin and gelatine hydrolysis, *β*-glucosidase and denitrification (API 20NE), as well as for alkaline phosphatase, esterase, esterase lipase, leucine arylamidase, trypsin, acid phosphatase, naphthol-AS-BI-phosphohydrolase, galactosidases, glucosidases, *N*-acetyl-*β*-glucosaminidase and *α*-mannosidase (API ZYM). Preferred compounds as a sole source of carbon are arabinose, dextrin, gentiobiose, glycogen, glucosamine, lyxose, malic acid, pectin, ribose and xylose. Acid is produced from 22 carbohydrates, including erythritol, d-arabinose, d-ribose, methyl-*β*-d-xylopyranoside, d-galactose, d-glucose, d-fructose, aesculin, salicin, d-cellobiose, d-maltose, d-lactose, d-melibiose, sucrose, starch and glycogen (API 50CHB/E). The genome size is 6.3 Mb, and the DNA G+C content is 72.1%.

The type strain is AC027S^T^ (=KCTC 59476^T^=JCM 37759^T^), isolated from root nodules of *Acacia gerrardii* grown in the Khurais desert, Saudi Arabia. The NCBI accession numbers of the 16S rRNA gene sequence and assembled genome are PV257722 and SAMN51309524, respectively.

## Supplementary material

10.1099/ijsem.0.007173 Uncited Fig. S1.

## Abbreviations

AAI: average amino acid identity
AF: alignment fraction
ANIb: average nucleotide identity based on blast
ANIb: average nucleotide identity based on blast
BCL: Bioscience Core Laboratory
dDDH: digital DNA–DNA hybridization
EPS: exopolysaccharide
gANI: genome-wide average nucleotide identity
LB: Luria–Bertani
LPSN: List of Prokaryotic Names with Standing in Nomenclature
MiSI: Microbial Species Identifier
NA: nutrient agar
NCBI: National Center for Biotechnology Information
PDA: potato dextrose agar
PGPT: plant growth-promoting traits
POCP: percentage of conserved proteins
R2A: Reasoner’s 2A agar
SEM: scanning electron microscopy
TSA: tryptic soy agar
TYGS: Type (Strain) Genome Server
