# Supplementary material for: Promicromonospora noduliphila sp. nov., a nodulation-enhancing actinobacterium isolated from the root nodules of grey-hair acacia planted in the Khurais desert, Saudi Arabia
Source: Int J Syst Evol Microbiol. 2026 Jun 9;76(6):007173. doi: 10.1099/ijsem.0.007173 (PMC13274763; doi:10.1099/ijsem.0.007173)
Supplement: Uncited Fig. S1. [file ijsem-76-07173-s001.pdf]

SUPPLEMENTARY MATERIAL of

Promicromonospora noduliphila sp. nov., a nodulation-enhancing actinobacterium isolated from the root nodules of grey-hair acacia planted in the Khurais desert, Saudi Arabia

Khulud Alghannam[1#], Grégoire Michoud[1,2#], Alan Barozzi[1], Sarah Al Romaih[1], Rawan Alhazmi[1], Bob Vernooij[1,3], Kennedy Odokonyero[1,3], Adair Gallo[1,3], Himanshu Mishra[1,3], Daniele Daffonchio[1,4], Ramona Marasco[1]*

[1]Biological and Environmental Sciences and Engineering Division (BESE), King Abdullah University of Science and Technology (KAUST), Thuwal, Saudi Arabia

[2]River Ecosystems Laboratory, Alpine and Polar Environmental Research Centre, ENAC, Ecole Polytechnique Fédérale de Lausanne, Sion, Switzerland

[3]Environmental Science and Engineering (EnSE) Program, King Abdullah University of Science and Technology (KAUST), 23955-6900, Thuwal, Kingdom of Saudi Arabia

[4]Department of Agriculture, Forestry and Food Sciences (DISAFA), University of Turin, Grugliasco, Turin, Italy

*Correspondence: Ramona Marasco, ramona.marasco@kaust.edu.sa

Supplementary Table 1. List of type strains within the genus *Promicromonospora* for which genomes are available and used as references for comparative analyses. The type strains selected for biochemical and physiological characterisation and comparison are in bold.

***Promicromonospora* species**	**Type strain ID**	**Genome acc. number**	**Total seq. length (bp)**	**Reference**
P. soli	**NEAU-GS50^{T}**	**GCA_014653785**	**5378526**	**[1]**
P. kroppenstedtii	RS16^{T}	GCF_044389675	6025996	[2]
P. iranensis	HM 792^{T}	GCF_031458275	5854223	[3]
P. vindobonensis	V-45^{T}	GCF_042685865	6643979	[4]
P. alba	1C-HV12^{T}	GCF_042654905	6432231	[5]
P. umidemergens	09-Be-007^{T}	GCF_024171995	6600894	[6]
P. aerolata	V-54A^{T}	GCF_042682255	6016682	[4]
P. thailandica	**S7F-02^{T}**	**GCF_024171955**	**5380599**	**[7]**
P. citrea	LL G-165^{T}	GCF_042660925	5147494	[8]
P. sukumoe	IFO 14650^{T}	GCF_014137995	6512079	[9]
P. xylanilytica	YIM 61515^{T}	GCA_054782895	5840549	[10]

Supplementary Table 2. Genome completeness and contamination values for the reference genomes used in the phylogenomic reconstruction.

Species name	Strain ID	Completeness	Contamination
Antribacter gilvus	CFH_30434	100	0.53
Antribacter soli	KLBMP_9083	99.86	2.01
Micrococcus luteus	NCTC_2665	100	0.05
Myceligenerans indicum	I2	99.9	0.01
Myceligenerans salitolerans	XHU_5031	99.98	1.05
Myceligenerans xiligouense	DSM_15700	99.98	0.04
Promicromonospora aerolata	CCM_7043	100	3.08
Promicromonospora alba	CGMCC_4.7283	99.72	0.59
Promicromonospora citrea	ATCC_15908	99.81	0.69
Promicromonospora iranensis	DSM_45554	100	1.14
Promicromonospora kroppenstedtii	DSM_19349	84.09	0.66
Promicromonospora soli	CGMCC_4.7398	100	0.43
Promicromonospora sukumoe	DSM_44121	98.81	0.8
Promicromonospora thailandica	DSM_26652	99.94	0.47
Promicromonospora umidemergens	DSM_22081	97.99	1.14
Promicromonospora vindobonensis	CCM_7044	98.51	1.56
Promicromonospora xylanilytica	JCM_19561	99.99	0.82
Xylanimonas allomyrinae	2JSPR-7	99.98	1.32
Xylanimonas cellulosilytica	DSM_15894	99.94	0.42
*Xylanimonas protaetiae_*	FW10M-9	99.92	0.64

Supplementary Table 3. List of respiratory quinones detected by HPLC-DAD/MS, all of which were menaquinones (MK). Percentages are reported for 1: AC027S^{T}, 2: AC027N, 3: *Promicromonospora thailandica* S7F-02^{T}, 4: *Promicromonospora soli* NEAU-GS50^{T}, 5: *Myceligenerans xiligouense* XLG9A10.2^{T}, 6: *Xylanimonas cellulosilytica* XIL07^{T}, 7: *Antribacter gilvus* CFH 30434^{T}. Analyses were carried out by DSMZ Services, Leibniz-Institut DSMZ - Deutsche Sammlung von Mikroorganismen und Zellkulturen GmbH, Braunschweig, Germany.

**Respiratory quinones (%)**	**1**	**2**	**3**	**4**	**5**	**6**	**7**
MK-7 H_4	1.2	0.9	3.0	4.1	7.7	1.0	2.2
MK-8 H_4	1.8	4.9	12.4	8.8	13.7	14.6	7.2
MK-8 H6	nd	nd	2.7	nd	0.5	nd	nd
MK-9	3.4	2.5	nd	nd	2.5	nd	nd
MK-9 H_2	8.0	10.6	0.5	3.9	6.4	nd	4.5
MK-9 H_4	70.9	66.6	62.6	82.8	30.1	82.3	86.1
MK-9 H_6	14.7	14.4	18.8	0.4	29	2.1	nd
MK-9 H_8	nd	nd	nd	nd	10.1	nd	nd

nd: not detected

Supplementary Figure 1. (**A** and **B**) Globose and palmate nodules collected from *Acacia* plants under a stereomicroscope before the sterilisation procedure, respectively. Bars' length 1 mm. (**C**) Two representative YEMA plates after three days of incubation at 28 °C; in the first plate, the resuspended solution obtained from the smashed nodule has been spread with a spatula, while in the second plate, portions of the smashed nodule have been directly distributed on the plate.

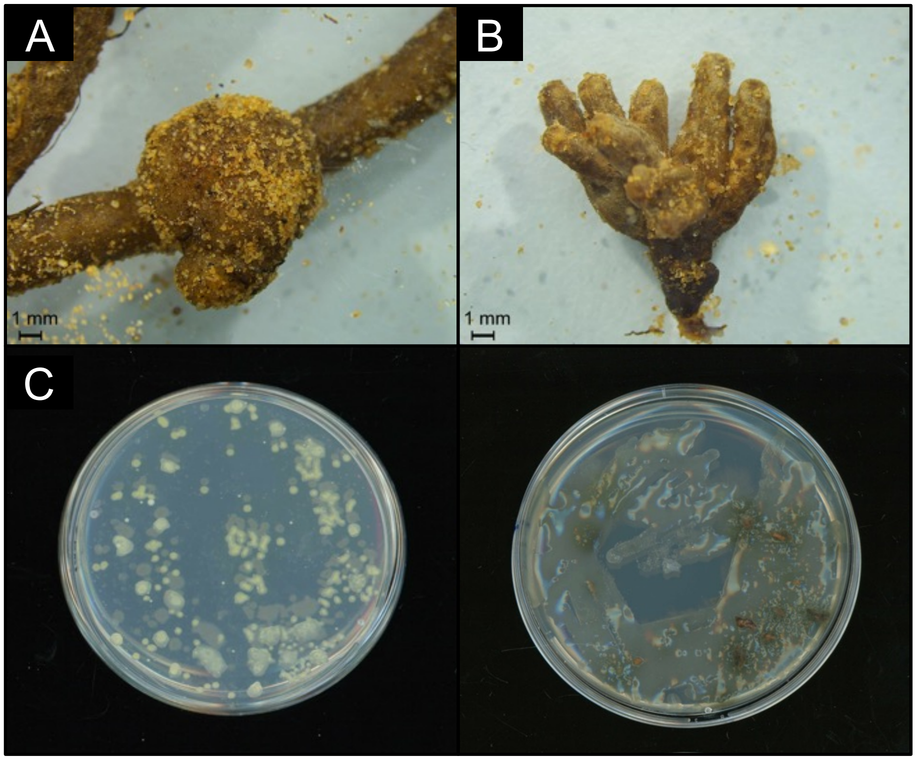

Supplementary Figure 2. Alignment of the 16S rRNA gene sequences in Geneious Prime from the five isolates, namely AC014S, AC026S, AC027S, AC025N, and AC027N. Percentage of identity (%) ranges from 99.5% to 100%; sequence size,1366-1373 bp.

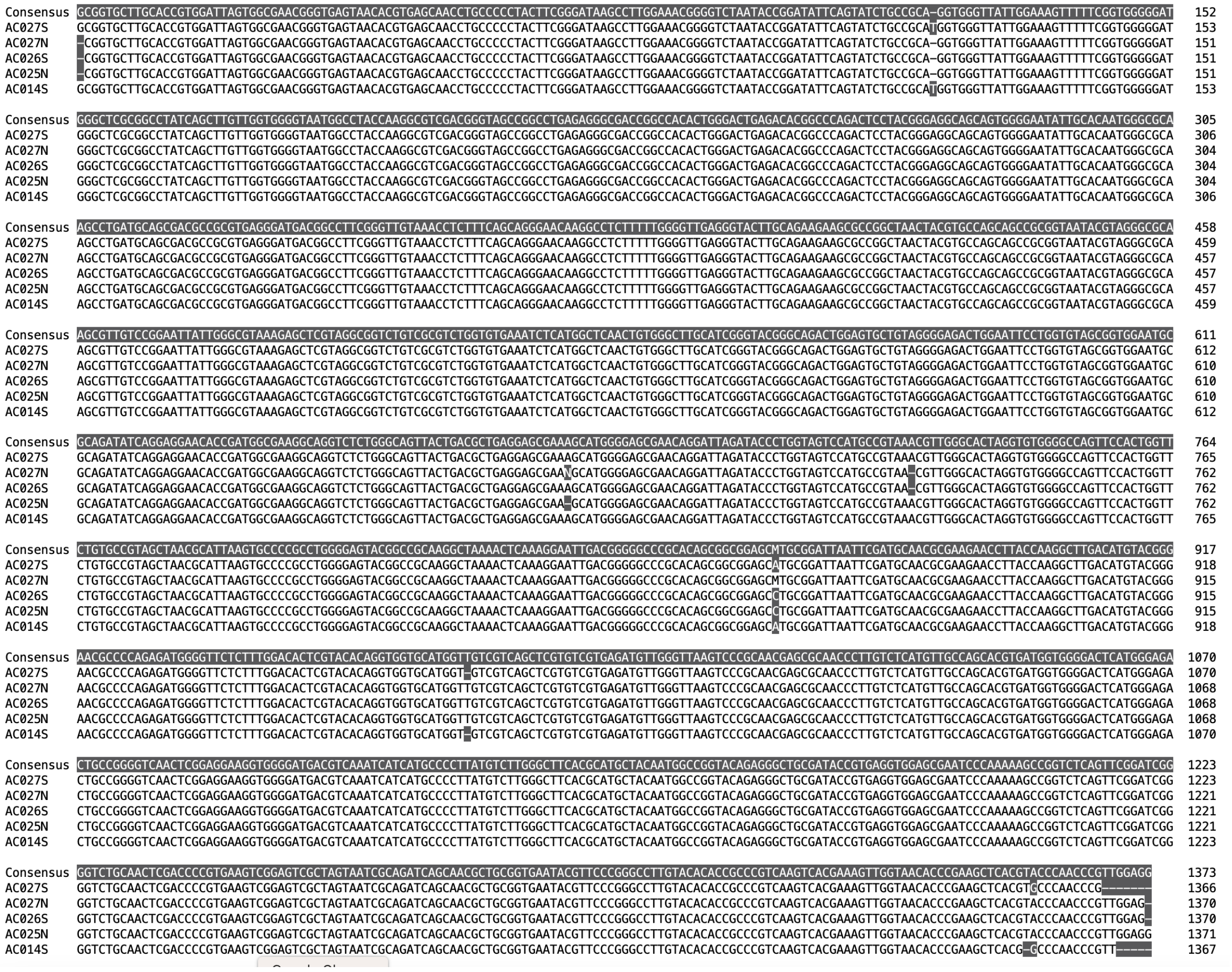

Supplementary Figure 3. The genome sequence data were uploaded to the Type (Strain) Genome Server (TYGS), a free bioinformatics platform available at https://tygs.dsmz.de, for whole-genome-based taxonomic analysis [11]. Information on nomenclature, synonymy and associated taxonomic literature was provided by TYGS's sister database, the List of Prokaryotic names with Standing in Nomenclature (LPSN, available at https://lpsn.dsmz.de) [12,13]. The results were provided by the TYGS on 2026-04-12. The resulting intergenomic distances were used to infer a balanced minimum evolution tree with branch support via FASTME 2.1.6.1, including SPR postprocessing [14]. Branch support was inferred from 100 pseudo-bootstrap replicates, and their lengths are scaled using the GBDP distance formula d5. The numbers above the branches are GBDP pseudo-bootstrap support values > 60%, with an average branch support of 86.8%. The trees were rooted at the midpoint [15] and visualised with PhyD3 [16].

Percent G+C	δ statistics	Genome size (in bp)	Number of proteins	SSU lengths (in bp)
70.79 - 73.57	0.117 - 0.286	4,957,134 - 6,643,979	4,551 - 6,686	1,331 - 1,521

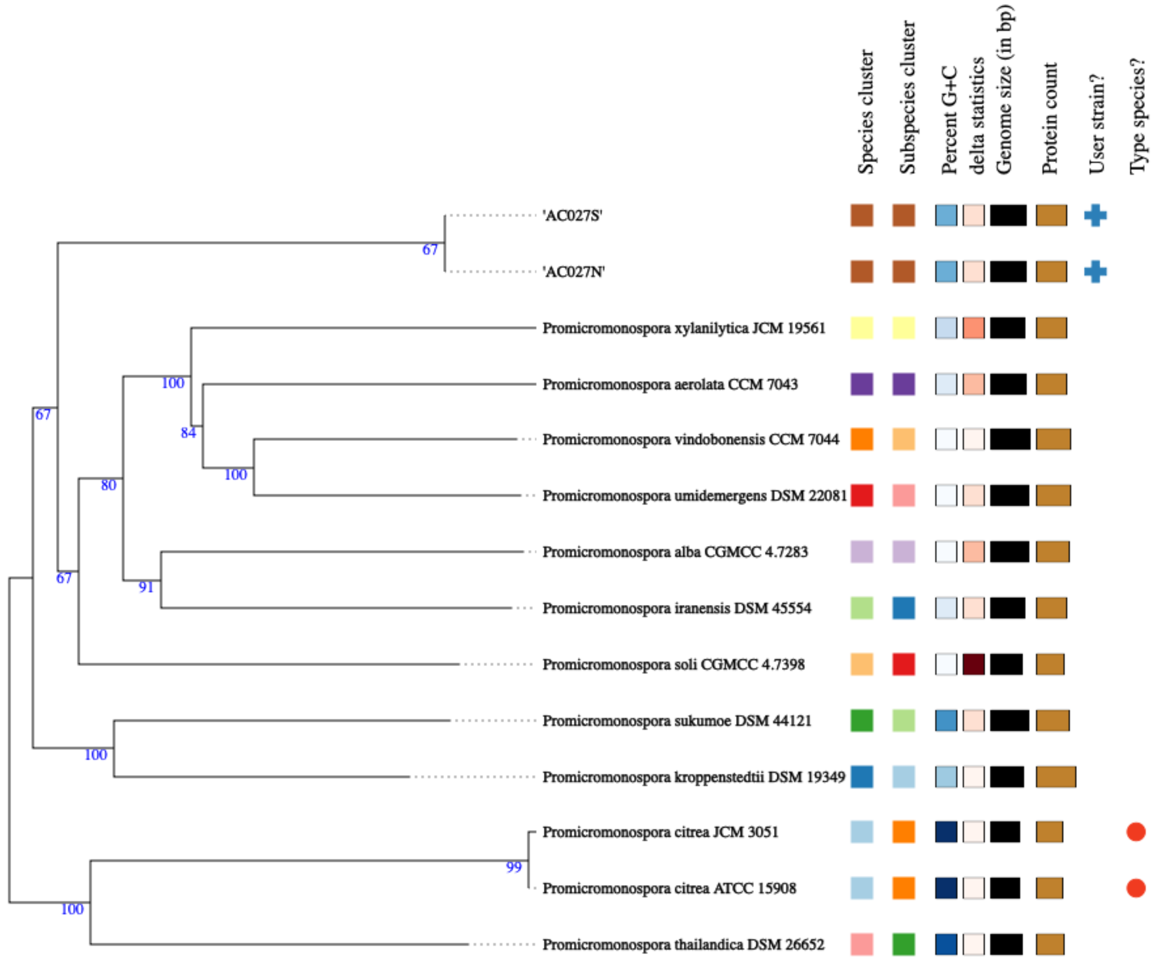

Supplementary Figure 4. Alignment in Geneious Prime of the 16S rRNA gene sequences retrieved from AC027S^{T} and AC025N genomes and those amplified via PCR.

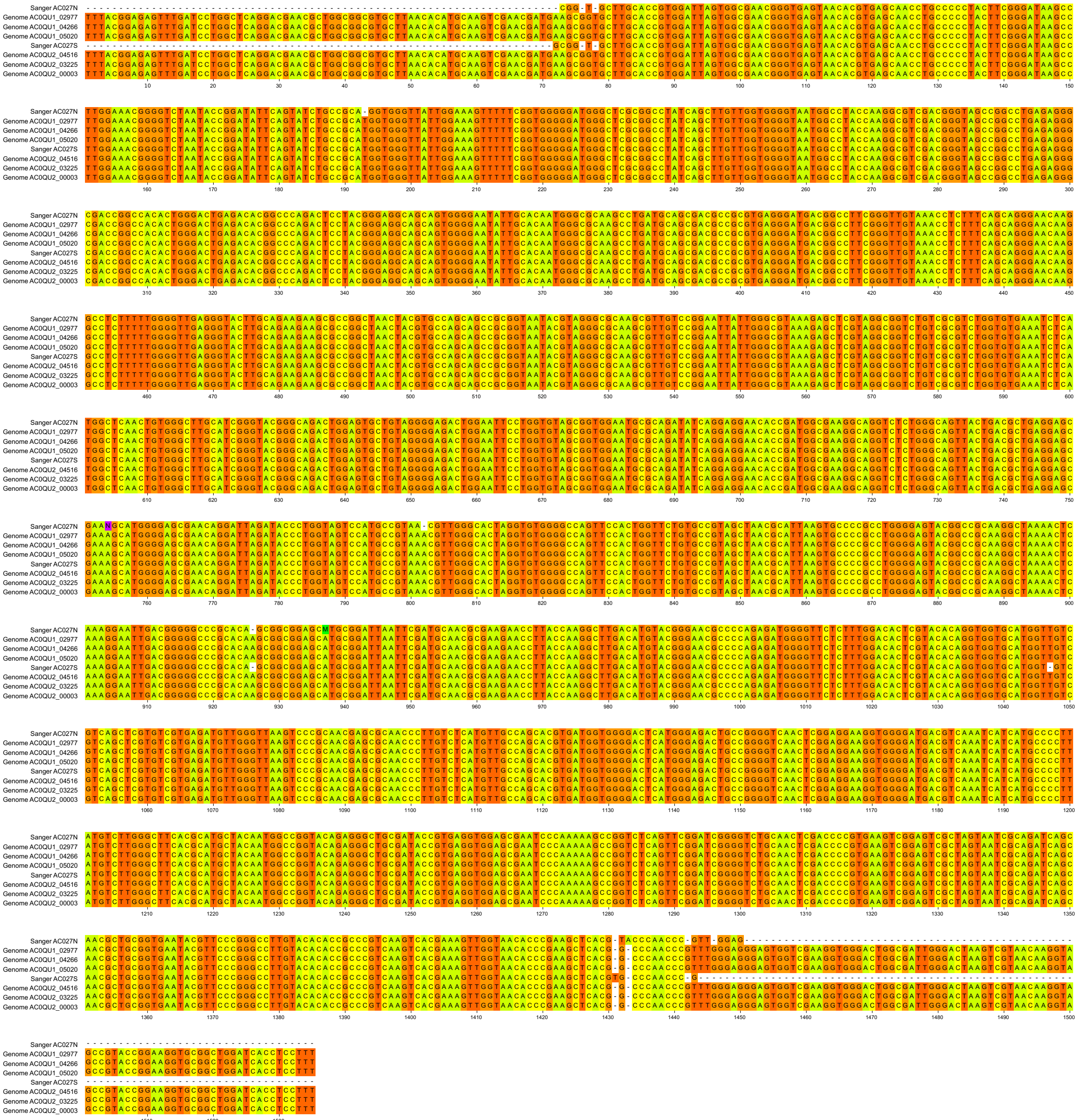

Supplementary Figure 5. Scanning electron micrographs of bacterial cells from AC027S^{T} (left column) and AC027N (right column) cultures grown on TSB medium at 28 °C. Different magnifications are reported for both strains.

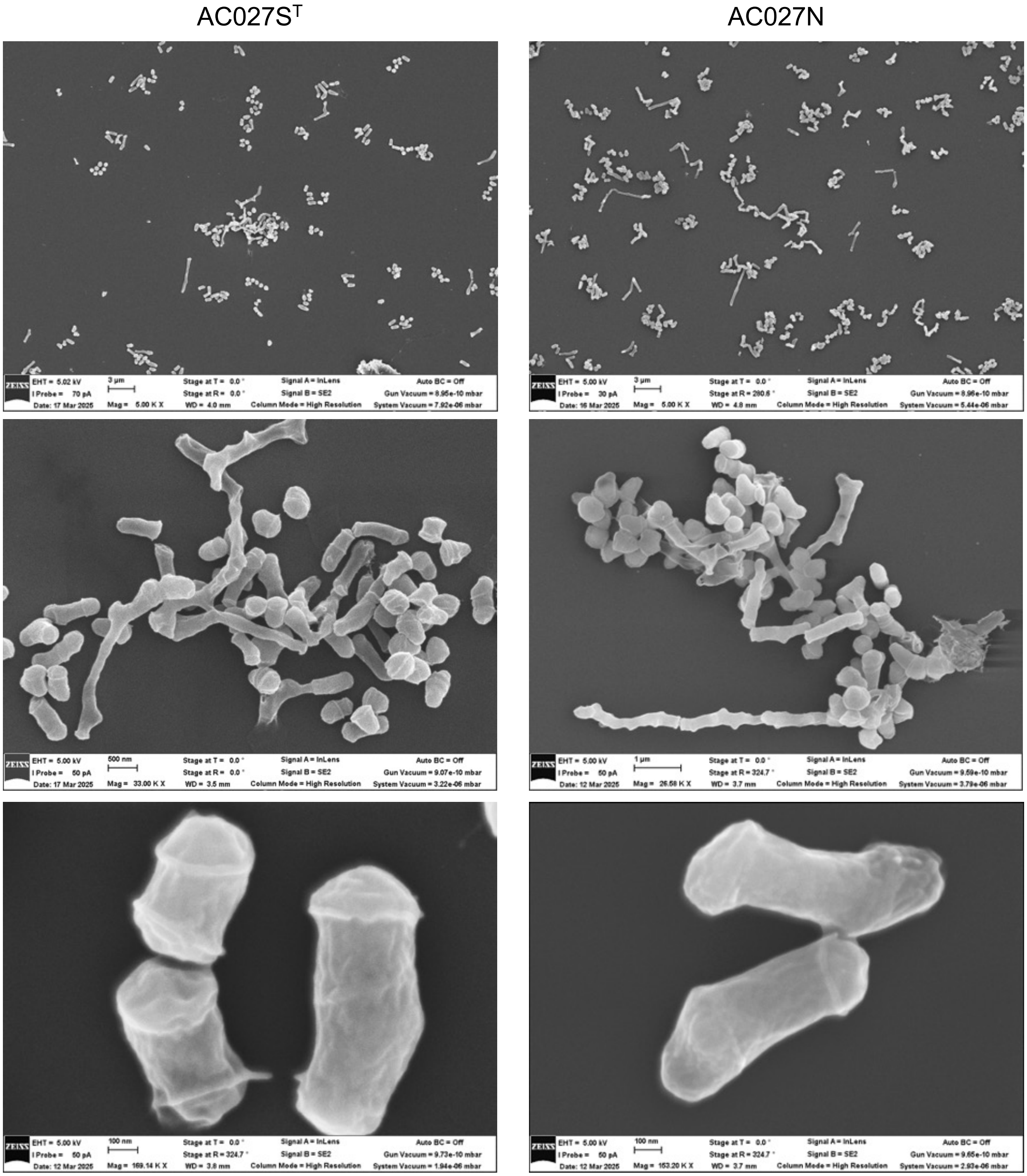

Supplementary Figure 6. Results of API50CH B/E test after 48 h of incubation.

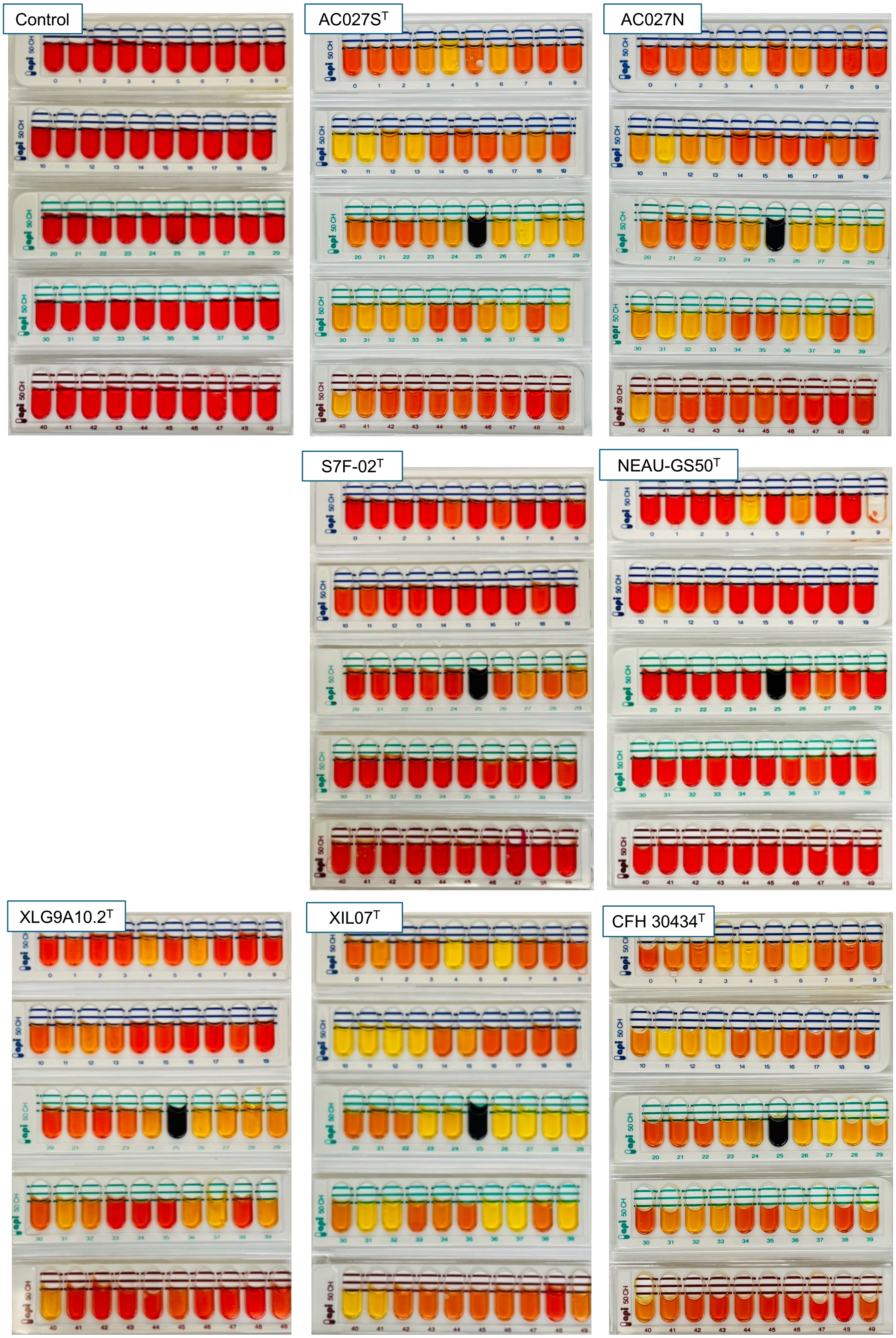

Supplementary Figure 7. GC-FID and -MS results of the cellular fatty acid conducted on the biomass of (**A**) AC027S^T, (**B**) AC027N, (**C**) *Promicromonospora thailandica* S7F-02^T, (**D**) *Promicromonospora soli* NEAU-GS50^T, (**E**) *Myceligenerans xiligouense* XLG9A10.2^T, (**F**) *Xylanimonas cellulosilytica* XIL07^T, (**G**) *Antribacter gilvus* CFH 30434^T. DSMZ Services carried out the analysis.

(**A**) AC027S^T

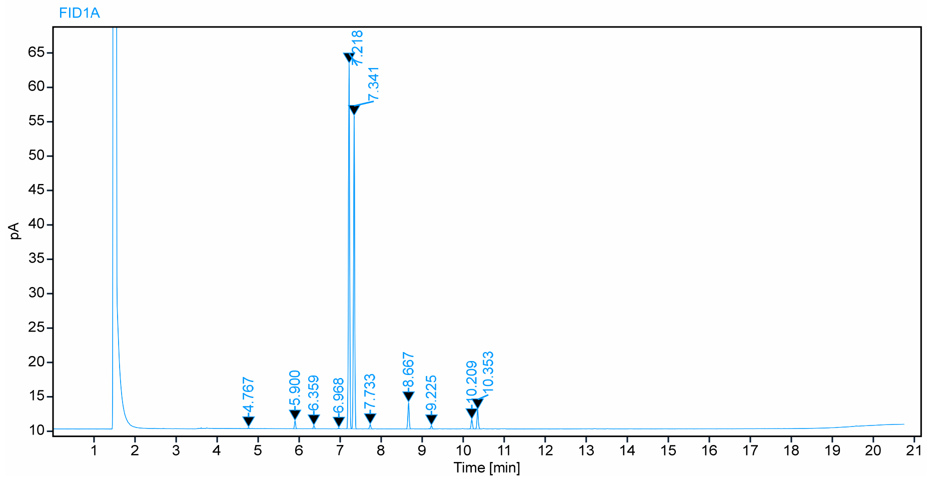

RT [min]	Rfact	Area	ECL	ΔECL	Percent	Naming MIDI/TSBA6	GC-MS
4.767	1.0425	0.3049	12.609	-0.005	0.1	13:0 iso	OK
5.900	1.0126	2.1968	13.614	-0.005	1.0	14:0 iso	OK
6.359	1.0222	0.9289	13.994	-0.006	0.4	14:0	OK
6.968	0.9812	0.2992	14.439	-0.001	0.1	15:1 iso G	Identified as 15:1 iso w10c
7.218	0.9864	109.5804	14.622	-0.001	48.6	15:0 iso	OK
7.341	0.9890	91.9282	14.712	-0.001	40.9	15:0 anteiso	OK
7.733	0.9972	1.3874	14.998	-0.002	0.6	15:0	OK
8.667	0.9686	8.4342	15.622	-0.005	3.7	16:0 iso	OK
9.225	0.9686	1.0857	15.995	-0.005	0.5	16:0	OK
10.209	0.9602	3.1717	16.625	-0.005	1.4	17:0 iso	OK
10.353	0.9623	6.3745	16.717	-0.006	2.8	17:0 anteiso	OK
		225.6919					

(**B**) AC027N

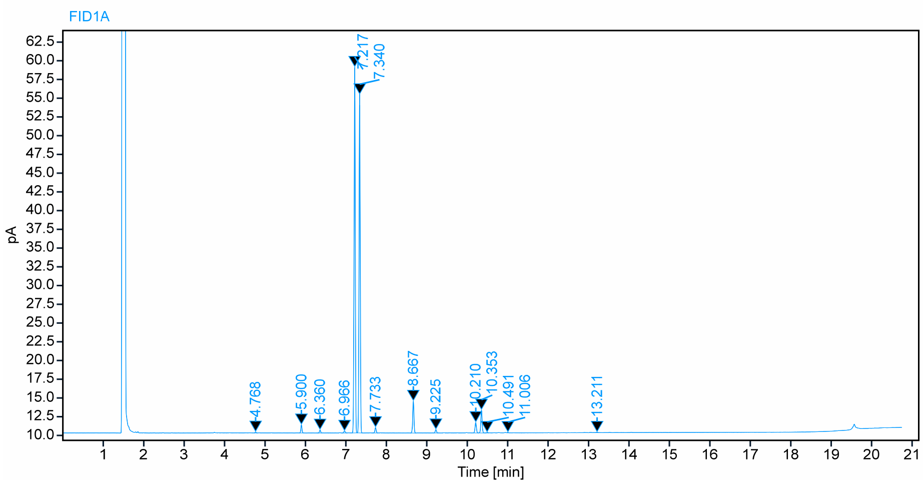

RT [min]	Rfact	Area	ECL	ΔECL	Percent	Naming MIDI/TSBA6	GC-MS
4.768	1.0426	0.2447	12.610	-0.004	0.1	13:0 iso	OK
5.900	1.0126	2.1006	13.614	-0.005	1.0	14:0 iso	OK
6.360	1.0222	0.8742	13.995	-0.005	0.4	14:0	OK
6.966	0.9812	0.6395	14.438	-0.002	0.3	15:1 iso G	Identified as 15:1 iso w10c
7.217	0.9864	101.1912	14.621	-0.002	46.3	15:0 iso	OK
7.340	0.9890	91.3397	14.711	-0.002	41.9	15:0 anteiso	OK
7.733	0.9972	1.5889	14.998	-0.002	0.7	15:0	OK
8.667	0.9686	9.3975	15.622	-0.005	4.2	16:0 iso	OK
9.225	0.9686	1.0362	15.995	-0.005	0.5	16:0	OK
10.210	0.9602	3.2165	16.626	-0.004	1.4	17:0 iso	OK
10.353	0.9623	7.0954	16.717	-0.006	3.2	17:0 anteiso	OK
10.491	0.9642	0.2483	16.806	-0.012		17:1 w7c	not confirmed
11.006	0.9449	0.1896	17.132				
13.211	0.9441	0.1864	18.512				
		**219.3487**					

(**C**) *P. thailandica* S7F-02[T]

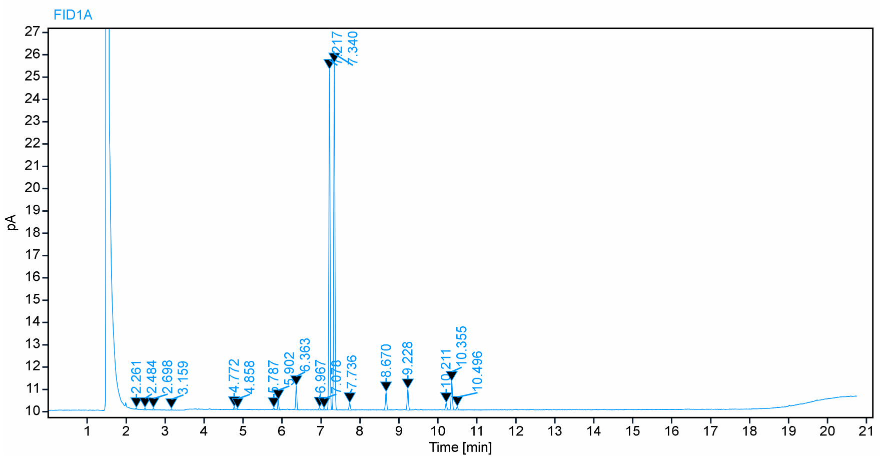

RT [min]	Rfact	Area	ECL	ΔECL	Percent	Naming MIDI/TSBA6	GC-MS
2.261		0.1636					
2.484		0.1506					
2.698		0.1525					
3.159	1.1428	0.0870	10.654				
4.772	1.0366	0.2329	12.611	-0.003	0.3	13:0 iso	OK
4.858	1.0396	0.0740	12.697	-0.005	0.1	13:0 anteiso	OK
5.787	1.0039	0.1968	13.518				
5.902	1.0062	0.8957	13.613	-0.006	1.2	14:0 iso	OK
6.363	1.0155	2.1220	13.995	-0.005	2.9	14:0	OK
6.967	0.9761	0.2462	14.436	-0.004	0.3	15:1 iso G	Identified as 15:1 iso w10c
7.078	0.9783	0.1497	14.517		0.2		Identified as 15:1 anteiso w10c
7.217	0.9810	31.2123	14.618	-0.005	41.3	15:0 iso	OK
7.340	0.9834	32.2977	14.708	-0.005	42.8	15:0 anteiso	OK
7.736	0.9912	0.6865	14.996	-0.004	0.9	15:0	OK
8.670	0.9653	1.8272	15.622	-0.005	2.4	16:0 iso	OK
9.228	0.9643	2.0604	15.995	-0.005	2.7	16:0	OK
10.211	0.9586	0.7316	16.625	-0.005	0.9	17:0 iso	OK
10.355	0.9607	2.9720	16.717	-0.006	3.9	17:0 anteiso	OK
10.496	0.9627	0.3738	16.807	-0.011		17:1 w7c	not confirmed
		**76.1658**					

(**D**) *P. soli* NEAU-GS50^T

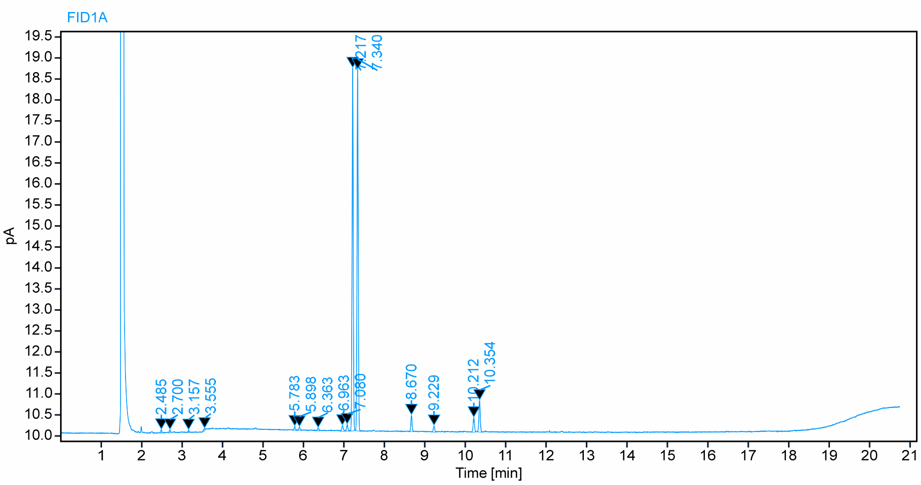

RT [min]	Rfact	Area	ECL	ΔECL	Percent	Naming MIDI/TSBA6	GC-MS
2.485		0.1427					
2.700		0.1472					
3.157	1.1425	0.1034	10.650				
3.555	1.0602	0.1300	11.239				
5.783	1.0038	0.2131	13.514				
5.898	1.0061	0.2007	13.610	-0.009	0.5	14:0 iso	OK
6.363	1.0155	0.1984	13.995	-0.005	0.5	14:0	OK
6.963	0.9760	0.3603	14.433	-0.007	0.9	15:1 iso G	Identified as 15:1 iso w10c
7.080	0.9783	0.3880	14.518	-0.009	1.0	15:1 anteiso A	Identified as 15:1 anteiso w10c
7.217	0.9810	17.6805	14.618	-0.005	43.6	15:0 iso	OK
7.340	0.9834	17.9624	14.708	-0.005	44.4	15:0 anteiso	OK
8.670	0.9653	0.8617	15.622	-0.005	2.1	16:0 iso	OK
9.229	0.9643	0.3561	15.996	-0.004	0.9	16:0	OK
10.212	0.9587	0.8333	16.625	-0.005	2.0	17:0 iso	OK
10.354	0.9607	1.7658	16.716	-0.007	4.3	17:0 anteiso	OK
		**41.0537**					

(**E**) *M. xiligouense* XLG9A10.2^T

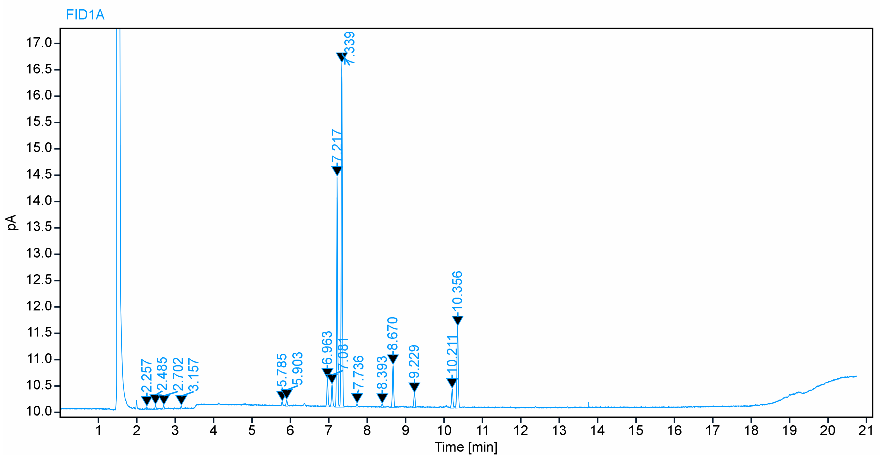

RT [min]	Rfact	Area	ECL	ΔECL	Percent	Naming MIDI/TSBA6	GC-MS
2.257		0.0785					
2.485		0.1582					
2.702		0.1244					
3.157	1.1425	0.1038	10.650				
5.785	1.0038	0.1745	13.516				
5.903	1.0062	0.2425	13.614	-0.005	0.8	14:0 iso	OK
6.963	0.9760	1.1240	14.433	-0.007	3.5	15:1 iso G	Identified as 15:1 iso w10c
7.081	0.9783	0.9690	14.519	-0.008	3.0	15:1 anteiso A	Identified as 15:1 anteiso w10c
7.217	0.9810	9.0509	14.618	-0.005	28.2	15:0 iso	OK
7.339	0.9834	13.5915	14.707	-0.006	42.4	15:0 anteiso	OK
7.736	0.9912	0.1560	14.996	-0.004	0.5	15:0	OK
8.393	0.9658	0.1552	15.436	-0.006	0.5	16:1 iso G	Identified as 16:1*
8.670	0.9653	1.7825	15.622	-0.005	5.5	16:0 iso	OK
9.229	0.9643	0.6302	15.996	-0.004	1.9	16:0	OK
10.211	0.9586	0.8877	16.625	-0.005	2.7	17:0 iso	OK
10.356	0.9607	3.6452	16.718	-0.005	11.1	17:0 anteiso	OK
		**32.513**					*too small for identification

(**F**) *X. cellulosilytica* XIL07^T

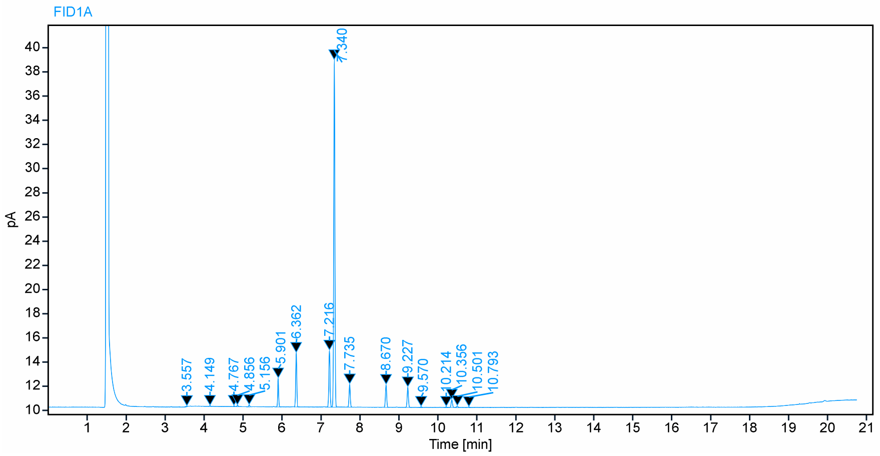

RT [min]	Rfact	Area	ECL	ΔECL	Percent	Naming MIDI/TSBA6	GC-MS
3.557	1.0765	0.1680	11.245				
4.149	1.1108	0.1506	11.991	-0.009	0.2	12:0	OK
4.767	1.0501	0.1356	12.609	-0.005	0.1	13:0 iso	OK
4.856	1.0535	0.2318	12.697	-0.005	0.3	13:0 anteiso	OK
5.156	1.0650	0.3125	12.996	-0.004	0.3	13:0	OK
5.901	1.0153	4.5582	13.614	-0.005	4.8	14:0 iso	OK
6.362	1.0264	8.8503	13.996	-0.004	9.4	14:0	OK
7.216	0.9856	9.7481	14.619	-0.004	10.0	15:0 iso	OK
7.340	0.9884	59.0988	14.710	-0.003	60.6	15:0 anteiso	OK
7.735	0.9975	4.0833	14.998	-0.002	4.2	15:0	OK
8.670	0.9651	4.0902	15.623	-0.004	4.1	16:0 iso	OK
9.227	0.9660	3.7380	15.995	-0.005	3.7	16:0	OK
9.570	0.9429	0.1647	16.215	-0.004		15:0 2OH	not confirmed
10.214	0.9538	0.1824	16.627	-0.003	0.2	17:0 iso	OK
10.356	0.9562	1.5444	16.718	-0.005	1.5	17:0 anteiso	OK
10.501	0.9587	0.3368	16.811	-0.007	0.3	17:1 w7c	OK
10.793	0.9636	0.0949	16.997	-0.003	0.1	17:0	OK
		**97.4886**					

(**G**) *A. gilvus* CFH 30434^T

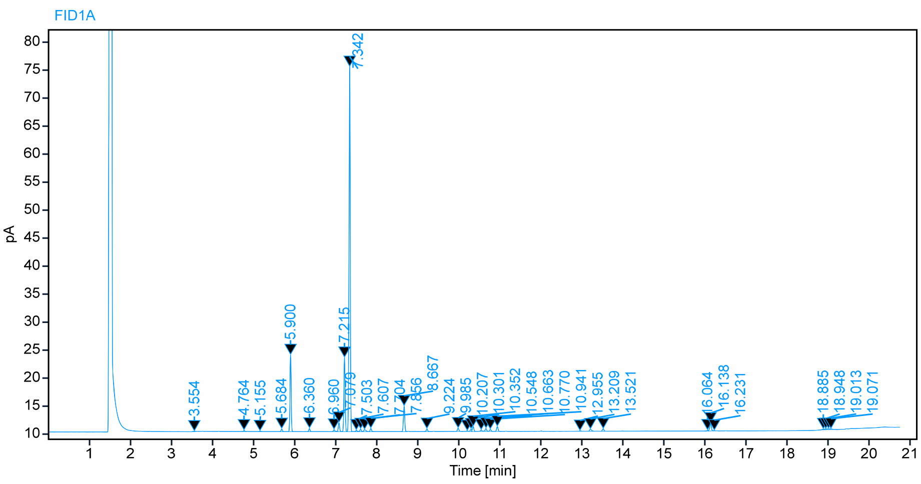

RT [min]	Rfact	Area	ECL	ΔECL	Percent	Naming MIDI/TSBA6	GC-MS
3.554	1.0848	0.1855	11.245				
4.764	1.0555	0.3867	12.609	-0.005	0.2	13:0 iso	OK
5.155	1.0730	0.2022	12.999	-0.001	0.1	13:0	OK
5.684	1.0146	0.9456	13.438		0.5		Identified as 14:1 iso w9c
5.900	1.0190	25.5645	13.617	-0.002	12.6	14:0 iso	OK
6.360	1.0283	1.1313	13.999	-0.001	0.6	14:0	OK
6.960	0.9715	0.8949	14.437	-0.003	0.4	15:1 iso G	Identified as 15:1 iso w10c
7.079	0.9765	3.3791	14.524	-0.003	1.6	15:1 anteiso A	Identified as 15:1 anteiso w10c
7.215	0.9822	27.6085	14.623	0.000	13.1	15:0 iso	OK
7.342	0.9875	134.8321	14.716	0.003	64.2	15:0 anteiso	OK
7.503	0.9943	0.5239	14.833				
7.607	0.9986	1.0942	14.909	0.006		15:1 w5c	not confirmed
7.704	1.0027	1.2186	14.980	-0.020	0.6	15:0	OK
7.856	0.9607	1.2824	15.083				not confirmed
8.667	0.9522	10.0462	15.626	-0.001	4.6	16:0 iso	OK
9.224	0.9463	1.1213	15.999	-0.001	0.5	16:0	OK
9.985	0.9494	1.5106	16.485	0.009		Summed Feature 4 (17:1 iso I/anteiso B)	not confirmed
10.207	0.9529	0.2667	16.627	-0.003	0.1	17:0 iso	OK
10.301	0.9544	0.9025	16.687				
10.352	0.9552	2.1204	16.719	-0.004	1.0	17:0 anteiso	OK
10.548	0.9582	0.6566	16.845				not confirmed
10.663	0.9600	1.4416	16.918	0.001		17:1 w5c	not confirmed
10.770	0.9617	0.9130	16.987	-0.013		17:0	not confirmed
10.941	0.9307	2.1685	17.094				
12.955	0.9308	0.2337	18.355				
13.209	0.9306	1.2134	18.514				
13.521	0.9303	1.0849	18.709				
16.064		0.3534					
16.138		3.7513					
16.231		0.2448					
18.885		0.3864					
18.948		0.5844					
19.013		0.4280					
19.071		0.3851					
		**222.9289**					

Supplementary Figure 8. Total polar lipids profile separation by two-dimensional TLC. Results are reported for (**A**) AC027S^T, (**B**) AC027N, (**C**) *Promicromonospora thailandica* S7F-02^T, (**D**) *Promicromonospora soli* NEAU-GS50^T, (**E**) *Myceligenerans xiligouense* XLG9A10.2^T, (**F**) *Xylanimonas cellulosilytica* XIL07^T, (**G**) *Antribacter gilvus* CFH 30434^T. DSMZ Services carried out the analysis.

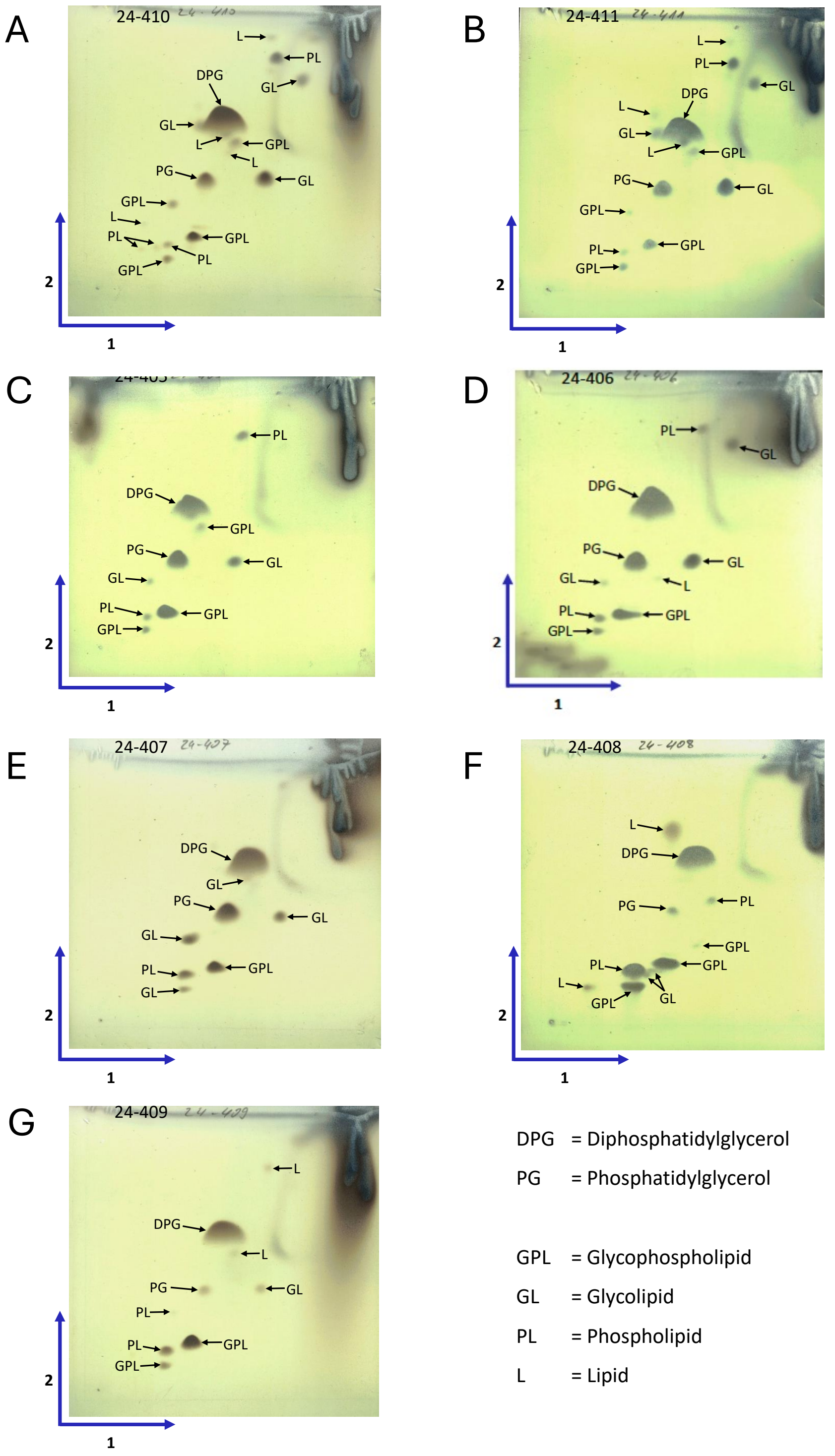

References

1. **Zheng W, Li D, Zhao J, Liu C, Zhao Y, *et al.*** *Promicromonospora soli* sp. nov., a novel actinomycete isolated from soil. *Int J Syst Evol Microbiol* 2017;67:3829–3833.
2. **Alonso-Vega P, Santamaria RI, Martinez-Molina E, Trujillo ME**. *Promicromonospora kroppenstedtii* sp. nov., isolated from sandy soil. *Int J Syst Evol Microbiol* 2008;58:1476–1481.
3. **Mohammadipanah F, Hamedi J, Spröer C, Montero-Calasanz M del C, Schumann P, *et al.*** *Promicromonospora iranensis* sp. nov., an actinobacterium isolated from rhizospheric soil. *Int J Syst Evol Microbiol* 2014;64:3314–3319.
4. **Busse H-J, Zlamala C, Buczolits S, Lubitz W, Kämpfer P, *et al.*** *Promicromonospora vindobonensis* sp. nov. and *Promicromonospora aerolata* sp. nov., isolated from the air in the medieval 'Virgilkapelle' in Vienna. *Int J Syst Evol Microbiol* 2003;53:1503–1507.
5. **Guo L, Liu C, Zhao J, Li C, Guo S, *et al.*** *Promicromonospora alba* sp. nov., an actinomycete isolated from the cuticle of *Camponotus japonicas* Mayr. *Int J Syst Evol Microbiol* 2016;66:1340–1345.
6. **Martin K, Schäfer J, Kämpfer P**. *Promicromonospora umidemergens* sp. nov., isolated from moisture from indoor wall material. *Int J Syst Evol Microbiol* 2010;60:537–541.
7. **Thawai C, Kudo T**. *Promicromonospora thailandica* sp. nov., isolated from marine sediment. *Int J Syst Evol Microbiol* 2012;62:2140–2144.
8. **Dmitrenok AS, Streshinskaya GM, Tul'skaya EM, Potekhina N V., Senchenkova SN, *et al.*** Pyruvylated cell wall glycopolymers of *Promicromonospora citrea* VKM A-665^{T} and *Promicromonospora* sp. VKM A-1028. *Carbohydr Res* 2017;449:134–142.
9. **Takahashi Y, Tanaka Y, Iwai Y, Ōmura S.** *Promicromonospora sukumoe* sp. nov., a new species of the *Actinomycetales*. J. Gen. Appl. Microbiol. 1987; 33:507-519.
10. **Qin S, Jiang JH, Klenk HP, Zhu WY, Zhao GZ, *et al.*** *Promicromonospora xylanilytica* sp. nov., an endophytic actinomycete isolated from surface-sterilized leaves of the medicinal plant Maytenus austroyunnanensis. *Int J Syst Evol Microbiol* 2012; 62:84-89.
11. **Meier-Kolthoff JP, Göker M.** TYGS is an automated high-throughput platform for state-of-the-art genome-based taxonomy. *Nat. Commun*. 2019;10: 2182. DOI: 10.1038/s41467-019-10210-3.
12. **Meier-Kolthoff JP, Sardà Carbasse J, Peinado-Olarte RL, Göker M.** TYGS and LPSN: a database tandem for fast and reliable genome-based classification and nomenclature of prokaryotes. *Nucleic Acid Res*. 2022;50: D801–D807. DOI: 10.1093/nar/gkab902.
13. **Freese HM, Meier-Kolthoff JP, Sardà Carbasse J, Afolayan AO, Göker M.** TYGS and LPSN in 2025: a Global Core Biodata Resource for genome-based classification and nomenclature of prokaryotes within DSMZ Digital Diversity. *Nucleic Acid Res*. 2025, gkaf1110. DOI: 10.1093/nar/gkaf1110.
14. **Lefort V, Desper R, Gascuel O.** FastME 2.0: A comprehensive, accurate, and fast distance-based phylogeny inference program. *Mol Biol Evol*. 2015;32: 2798–2800. DOI: 10.1093/molbev/msv150.

15. **Farris JS.** Estimating phylogenetic trees from distance matrices. *Am Nat*. 1972;106: 645–667.

16. **Kreft L, Botzki A, Coppens F, Vandepoele K, Van Bel M.** PhyD3: A phylogenetic tree viewer with extended phyloXML support for functional genomics data visualization. Bioinformatics. 2017;33: 2946–2947. DOI: 10.1093/bioinformatics/btx324.
